# From pathogenic carriers to therapeutic hope: the dual role and translational prospects of exosomes in diabetic kidney disease

**DOI:** 10.3389/fendo.2026.1831272

**Published:** 2026-07-02

**Authors:** Fen Li, Ruyi Zhong, Qing Qiu

**Affiliations:** Department of Endocrinology and Metabolism, The Liuyang People’s Hospital, Liuyang, China

**Keywords:** biomarkers, diabetic kidney disease, exosomes, intercellular communication, meta-analysis, translation

## Abstract

Diabetic kidney disease is one of the most serious microvascular complications of diabetes. Its pathogenesis is complex, and methods for early diagnosis and intervention are limited, posing major challenges in clinical practice. Exosomes, as key carriers of intercellular communication, are involved in various physiological and pathological processes, such as immune regulation, tumor metastasis, and tissue repair. The regulation of their release is closely related to disease progression and has become a hotspot in research on disease diagnosis and treatment. In diabetic kidney disease, exosomes play a dual role: on the one hand, “pathogenic exosomes”, produced under pathological stimuli such as hyperglycemia, can transmit harmful substances, exacerbating kidney damage, through inflammation and fibrosis; on the other hand, “therapeutic exosomes” that are derived from stem cells or engineered otherwise, have the potential to protect and repair the kidneys. Previous reviews have predominantly concentrated on the biomarker or therapeutic potential of exosomes in diabetic nephropathy. This paper, however, systematically integrates the multifaceted roles of exosomes in the pathogenesis of diabetic nephropathy and analyses the specific application evidence of exosome-derived biomarkers across various clinical functions. It highlights the dynamic changes in exosomes during disease progression and discusses targeted intervention strategies. Additionally, it examines the opportunities and challenges in clinical translation, aiming to offer novel insights and strategies for the precise diagnosis and targeted treatment of this condition.

## 1 Introduction

Diabetic kidney disease (DKD) is among the most severe microvascular complications of diabetes mellitus (DM) and is a leading cause of end-stage renal disease (ESRD) (1). Its pathological process is extremely complex and involves the interplay of multiple mechanisms, such as metabolic abnormalities, chronic inflammation, oxidative stress, immune dysregulation, and fibrosis (2). Prolonged hyperglycemia not only directly damages intrinsic renal cells but also triggers a cascade of reactions, including lipid metabolism disorders, mitochondrial dysfunction, endoplasmic reticulum stress, and overactivation of the renin–angiotensin– aldosterone system (RAAS) (3).

Although traditional therapies, such as strict glycemic control and the use of angiotensin-converting enzyme inhibitors (ACEIs) or angiotensin II receptor blockers (ARBs), can delay the progression of DKD to some extent, they often fail to completely prevent its eventual progression into end-stage renal disease (4). In recent years, novel antidiabetic medications, such as sodium–glucose cotransporter 2 (SGLT2) inhibitors and glucagon-like peptide-1 (GLP-1) receptor agonists, have been shown to have cardiorenal protective effects independent of their glucose-lowering effects, suggesting their potential for the treatment of DKD (5, 6). However, given the highly complex pathogenesis of DKD, which involves interactions among genetic, epigenetic, and environmental factors, further research into novel pathogenic mechanisms and intervention targets is urgently needed to develop more precise and effective diagnostic and therapeutic approaches (7).

As key carriers of intercellular signaling, exosomes are emerging as a focal point in DKD research (8). Exosomes are membrane-bound microvesicles secreted by cells and are widely distributed in the extracellular matrix and various body fluids. They carry a variety of bioactive molecules (9), such as proteins, lipids, mRNAs, and microRNAs (miRNAs). They can transmit information to nearby or distant target cells, thereby playing critical regulatory roles in physiological and pathological processes (10). In the context of DKD, the role of exosomes exhibits a clear duality: on the one hand, exosomes derived from damaged intrinsic kidney cells (such as podocytes, renal tubular epithelial cells, and mesangial cells) or circulating cells in a hyperglycemic environment can transmit proinflammatory, profibrotic, and proapoptotic signals, thereby promoting the progression of glomerulosclerosis and tubulointerstitial fibrosis (11); on the other hand, mesenchymal stem cells (MSCs) or their modified exosomes can deliver bioactive molecules such as miRNAs and proteins to regulate cellular stress states, promote intercellular communication, and accelerate tissue repair processes (12).

Research on the role of exosomes in diabetic nephropathy has expanded significantly; however, several key knowledge gaps and unresolved challenges persist. Firstly, the absence of a “gold standard” for separation and purification techniques remains the primary obstacle to clinical translation. Secondly, the high heterogeneity of exosomes and their dynamic changes at various stages of DKD have yet to be thoroughly analyzed. Most existing biomarker studies rely on small sample sizes and lack large-scale, multi-center validation. Furthermore, the application and translation of treatments encounter significant bottlenecks. Although engineered exosomes have proven effective as drug delivery carriers in animal models, their large-scale production, quality control, and targeted modification technologies are still not fully developed. Therefore, in-depth research into the dual role of exosomes in DKD not only elucidates new mechanisms of disease progression but also offers novel insights for the development of biomarkers and targeted therapeutic strategies.

## 2 Biological characteristics of exosomes and their communication network in the kidney

### 2.1 Biosynthesis, composition, and release regulation of exosomes

**Figure 1:**
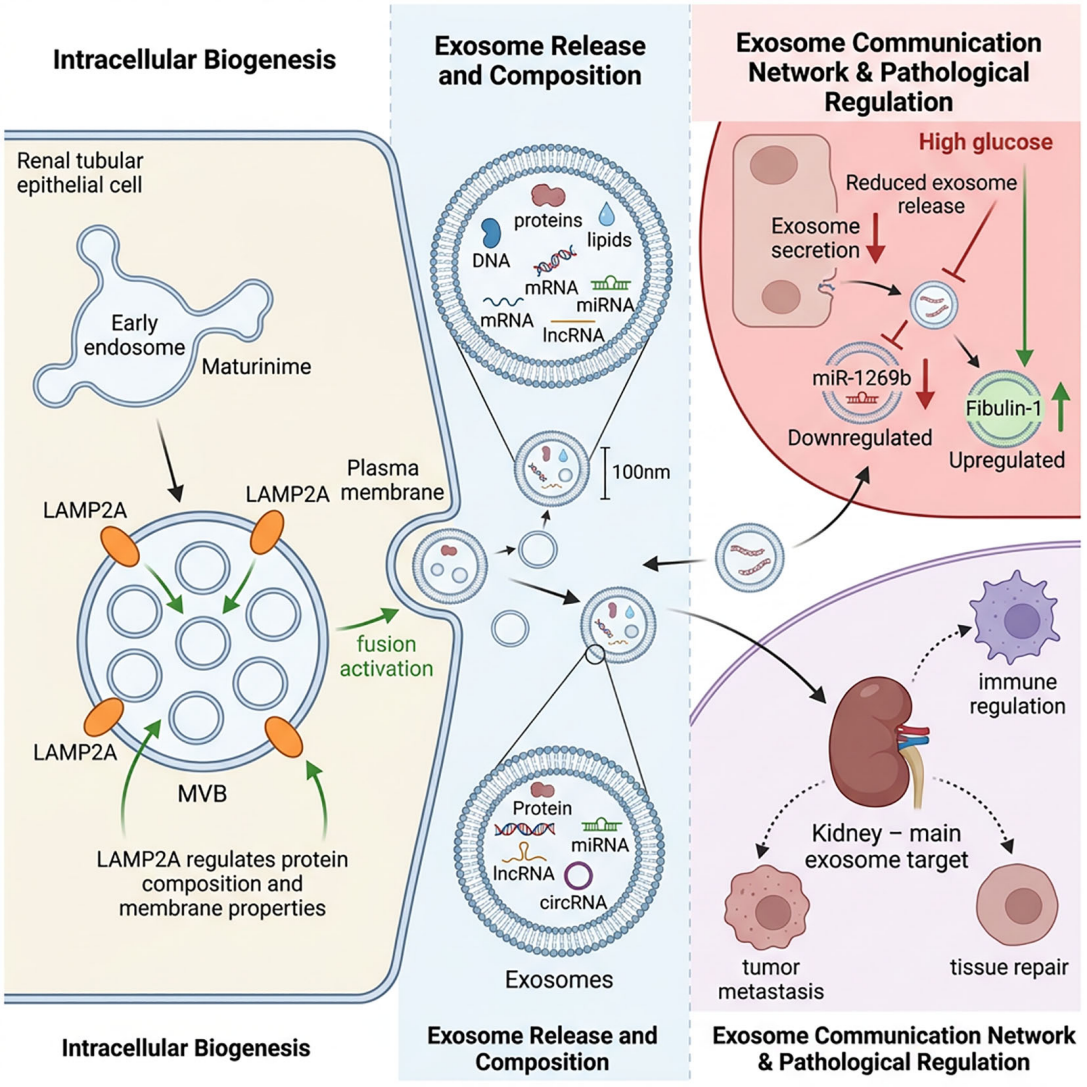
Biosynthesis, composition, and release regulation of exosomes. Exosomes originate from early endosomes, which mature to form multivesicular bodies (MVBs). Within this pathway, LAMP2A regulates the composition and membrane properties of MVB proteins, thereby mediating the fusion of MVBs with lysosomes. Mature exosomes encapsulate proteins, DNA, RNA (mRNA, lncRNA, miRNA, circRNA), and lipids. Upon release, exosomes primarily target the kidneys, contributing to tissue repair and tumor metastasis. In the hyperglycemic environment of diabetic nephropathy, exosome release is reduced, miR-1269b expression is downregulated, and some lncRNAs are upregulated, suggesting that the exosomal communication network plays a significant role in the pathological regulation of diabetic nephropathy.

Extracellular nano-sized vesicles, known as exosomes, are secreted by cells through a precisely controlled biosynthesis process (13). Exosome formation begins with cellular endocytosis. Upon the fusion of multivesicular bodies with the plasma membrane, the intraluminal vesicles are released, thereby constituting exosomes (14). Lysosome-associated membrane protein 2A (LAMP2A) is crucial in regulating protein composition and membrane properties, influencing exosome biosynthesis (15). Exosomes, encased in phospholipid bilayers, contain proteins, lipids, DNA, and various RNAs, including mRNA, miRNA, lncRNA, and circRNA (13, 16). They participate in physiological and pathological processes such as immune regulation, tumor metastasis, and tissue repair. The regulation of their release is intricately linked to disease progression (**Figure 1**). Exosomes form a complex communication network, with the kidney being a primary target organ (17). Hyperglycemia and metabolic stress profoundly alter exosome biogenesis and cargo sorting, a distinctive pathological feature of DKD. These conditions prompt renal tubular epithelial cells and podocytes to release aberrant exosomes whose altered cargo (e.g., miR-200a-3p, CKAP4, miR-3147) contributes to inflammation, fibrosis and tubular injury in DKD (**Figure 1**). For instance, extracellular vesicles from mesangial cells exposed to high glucose induced damage in healthy podocytes (18). Likewise, vesicles from CD4+T cells cultured under sustained high glucose exacerbated injury to renal tubular epithelial cells (19). Metabolic stress also modified vesicle cargo: miR-3147 in mesangial-cell vesicles promoted cell proliferation (20). Collectively, these processes establish a vicious cycle of inflammation and fibrosis (21), thereby identifying critical targets for exosome-based diagnostic and therapeutic strategies in DKD.

### 2.2 The communication network of exosomes

As key extracellular vesicles, exosomes establish a multilayered and dynamic intercellular communication network within the structurally complex and functionally sophisticated kidney (22). Once taken up by target cells, exosomes deliver functional molecules (such as microRNAs, proteins, and lipids) into these cells, thereby regulating gene expression and a range of biological processes, including proliferation, differentiation, migration, apoptosis, and inflammatory responses (16). Under pathological conditions, the renal exosome communication network undergoes significant alterations, contributing to disease onset and progression. In hypertensive patients with proteinuria, urine- and plasma-derived exosomes exhibit distinct miRNA profiles, particularly with the downregulation of miR-26a in both types of exosomes. This miRNA is a major regulator of the TGF-β signaling pathway and plays a vital role in podocyte injury (23).

**Figure 2:**
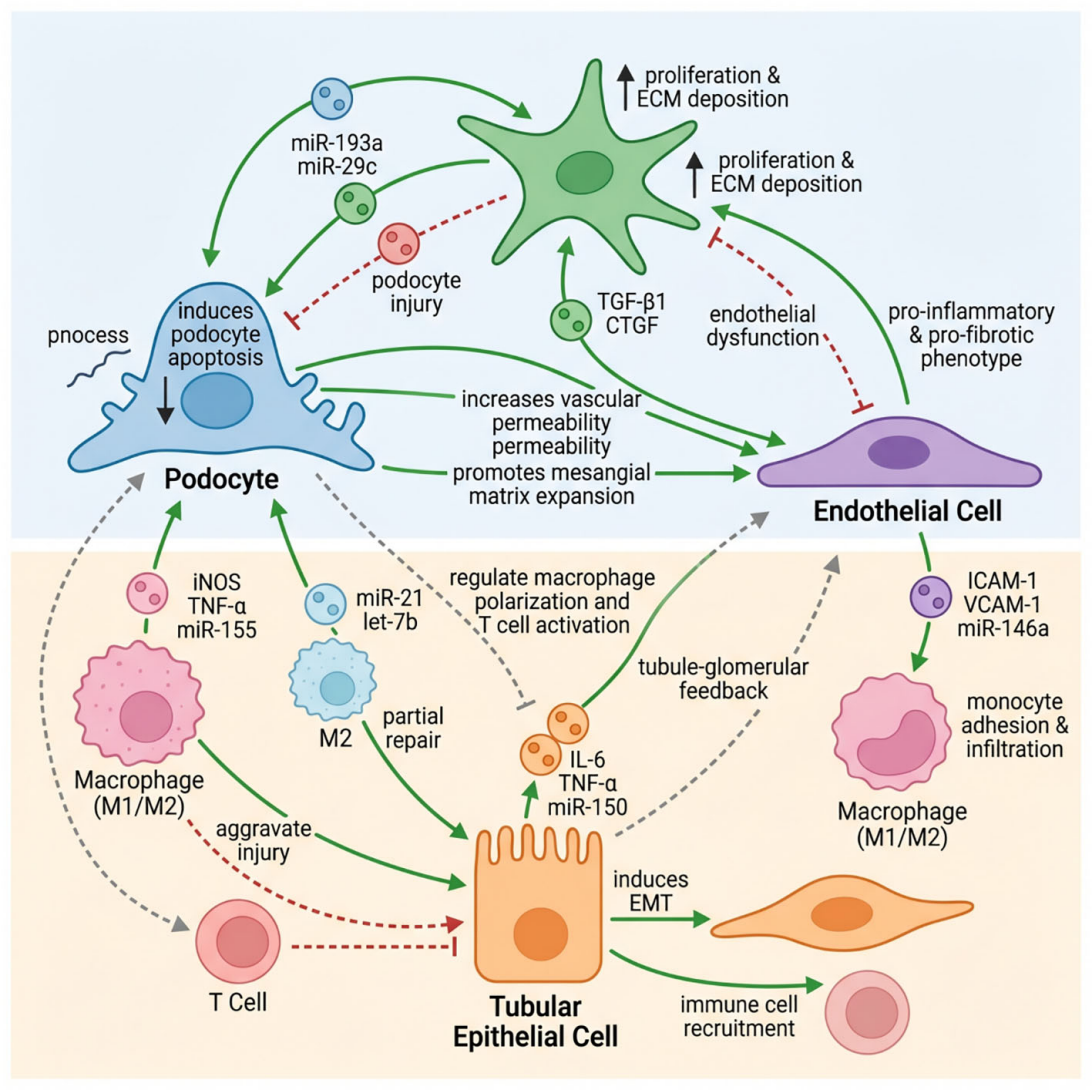
In the microenvironment of DKD, exosomes mediate a complex communication network among podocytes, mesangial cells, renal tubular epithelial cells, endothelial cells, and immune cells. Podocytes stimulated by high glucose/AGEs release exosomes enriched with dysregulated miRNAs such as miR-193a and miR-29c, triggering autocrine apoptosis and influencing endothelial cells and mesangial cells, thereby promoting increased vascular permeability and mesangial matrix expansion. Exosomes derived from mesangial cells exhibit elevated levels of TGF-β1 and CTGF, which not only promote their own proliferation and ECM deposition but can also be taken up by podocytes and endothelial cells, exacerbating podocyte injury and endothelial dysfunction. Injured renal tubular epithelial cells release exosomes containing IL-6, TNF-α, and miR-150, inducing EMT in adjacent epithelial cells and immune cell chemotaxis and infiltration, while also exerting a retrograde effect on glomerular cells, forming a tubule-glomerular feedback loop. Exosomes derived from endothelial cells are enriched in ICAM-1, VCAM-1, and miR-146a, promoting monocyte adhesion and infiltration, and are taken up by podocytes and mesangial cells, inducing a pro-inflammatory and pro-fibrotic phenotype. Within the immune cell compartment, particularly macrophages, M1-type exosomes encapsulate iNOS, TNF-α, and miR-155, exacerbating injury to podocytes and renal tubular epithelial cells, while M2-type exosomes may exert partial reparative effects through miR-21/let-7b. Additionally, exosomes from renal intrinsic cells can reciprocally regulate macrophage polarization and T cell activation.

In DKD, exosome-mediated intercellular communication facilitates intricate crosstalk among various cell types within the glomerulus and tubulointerstitium. Exosomes, along with their molecular cargoes like proteins and microRNAs, enable interactions among podocytes, mesangial cells, tubular epithelial cells, endothelial cells, and immune cells, thereby affecting disease progression (**Figure 2**).

Podocytes play a crucial role in maintaining the glomerular filtration barrier and are compromised early in DKD. Urinary exosomes originating from podocytes mirror their pathological condition. These exosomes carry microRNAs, such as miR-155, miR-21, and miR-29, as well as proteins like Regucalcin and Elf3, which are implicated in inflammation, oxidative stress, and fibrosis (24). They can be internalized by neighboring mesangial or endothelial cells, transmitting pro-fibrotic signals. Conversely, urinary migrasomes, a novel type of extracellular vesicle primarily from podocytes and macrophages, can trigger an inflammatory response in renal tubular epithelial cells via the TLR4-NF-κB pathway (25). The interaction between renal tubular epithelial cells and immune cells is also pivotal in DKD fibrosis. Single-cell transcriptome sequencing has shown that CD68^+^VEGF^+^TGF-β1^+^ macrophages enhance NUAK1 expression in renal tubular epithelial cells, promoting fibrosis (26). In contrast, MSC-EVs deliver Nedd4, which degrades NUAK1, thereby exerting an anti-fibrotic effect (26). Exosomes may also mediate interactions between immune and endothelial cells. Research indicates that urinary migrasomes contain ITGB3 and FGB, which are linked to inflammatory and fibrotic pathways. Levels of NLRC4, ITGB3, and FGB are significantly elevated in the renal tissues of DKD patients (25). Although direct evidence is limited, the presence of immune cell cargo in urinary exosomes suggests that exosomes from monocytes/macrophages might influence endothelial cell function and contribute to glomerular microvascular injury. Moreover, MSC-EVs can enhance renal function through anti-inflammatory, anti-fibrotic, and autophagy- restoring mechanisms, while modulating T cell and macrophage phenotypes, thereby indirectly safeguarding glomerular and renal tubular cells (27). These insights offer new perspectives on the pathological mechanisms of DKD and the development of exosome-based targeted therapies. However, systematic research on exosome communication between mesangial and endothelial cells remains scarce, presenting a promising area for future exploration.

Exosome-mediated communication is not limited to the local kidney but also involves long-distance communication between organs. Studies have confirmed that in pancreatic ductal adenocarcinoma models, exosomes derived from cancer cells can establish specific communication pathways with distant organs, such as the kidneys and lungs (17). This indicates that the exosome-mediated communication network not only coordinates the dialogue between different cell types within the kidney but also mediates long-distance interactions between the kidney and other organ systems throughout the body, which is crucial for maintaining renal homeostasis and responses to injury and disease.

## 3 Pathogenic exosomes: key mediators driving the progression of diabetic kidney disease

### 3.1 High glucose, insulin resistance, lipotoxicity, and metabolic memory-induced kidney cell-derived exosomes

**Figure 3:**
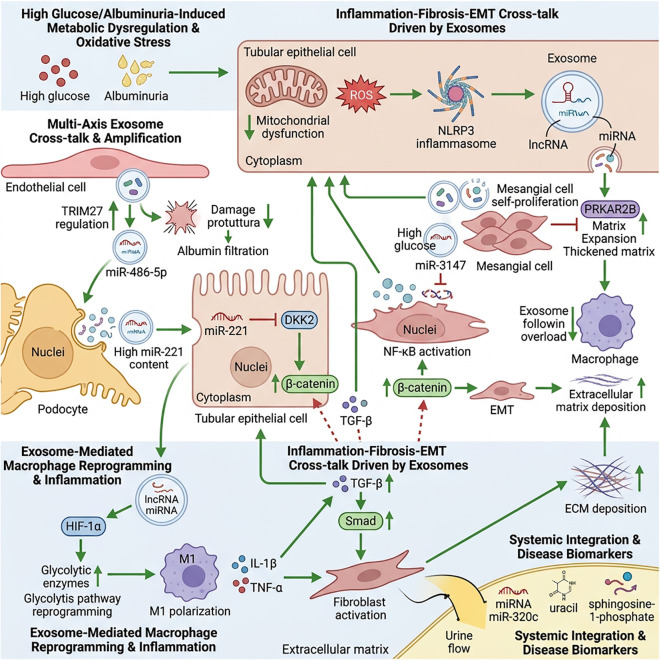
Elucidating the mechanisms of pathogenic exosomes driving the progression of DKD. Within a microenvironment characterized by hyperglycemia and albuminuria, glomerular endothelial cells, podocytes, and mesangial cells release pathogenic exosomes enriched with molecules such as miR-486-5p, miR-221, and miR-3147, which amplify the inflammatory response by inducing HIF-1α-mediated glycolytic reprogramming and M1 polarization of macrophages. Concurrently, by mediating ROS accumulation, mitochondrial dysfunction, and the activation of the NF-κB/β-catenin/TGF-β signaling axis, these exosomes promote mesangial cell proliferation, matrix expansion, and EMT of renal tubular epithelial cells. The TGF-β/Smad pathway further integrates the inflammation-fibrosis-EMT interactive network, ultimately establishing a comprehensive mechanistic framework and driving the progression of diabetic nephropathy. Collectively, these pathways offer a molecular foundation for the identification of novel DKD biomarkers.

Exosomes, a specific subtype of extracellular vesicles, play a pivotal role in intercellular communication during the onset and progression of DKD. The renal injury mechanism they mediate involves multiple pathways. The four primary metabolic abnormalities—hyperglycemia, insulin resistance, lipotoxicity, and metabolic memory-collectively contribute to and worsen the kidney’s pathological process. They achieve this by influencing exosome release, content packaging, and target cell response (**Figure 3**).

A high-glucose environment significantly alters the characteristics of exosomes released by renal cells, particularly podocytes and renal tubular epithelial cells. The exosomes secreted by these cells and the substances they carry undergo significant changes. These exosomes act as “messengers”, carrying harmful substances and playing a crucial role in transmitting signals during the development of fibrosis in DKD. It is well documented that under high-glucose stimulation, the number of exosomes released by renal tubular epithelial cells decreases, but their profibrotic capacity is significantly enhanced (28). Mechanistically, exosomes released by high-glucose-treated macrophages are enriched in inflammatory factors, such as interleukin-1β and inducible nitric oxide synthase. These factors promote renal inflammatory responses in both *in vivo* and *in vitro* models, as well as mesangial expansion by activating nuclear factor-κB p65 signaling, thereby exacerbating kidney injury (29).

Furthermore, under high-glucose stimulation, proximal renal tubular epithelial cells can secrete exosomes enriched with miR-92a-1-5p. After being taken up by glomerular mesangial cells, these exosomes induce endoplasmic reticulum stress by downregulating reticulocalbin-3, thereby promoting mesangial cell trans- differentiation into myofibroblasts and glomerulosclerosis (30). Similarly, lncRNAs derived from exosomes of high-glucose-treated macrophages, which are associated with signaling pathways such as MAPK, can downregulate E-cadherin expression in renal tubular epithelial cells. Concurrently, they can upregulate the expression of α-smooth muscle actin and fibronectin, thereby inducing epithelial–mesenchymal transition (EMT) (31). These findings indicate that in a high-glucose environment, exosomes released by renal cells act as carriers of harmful molecules. They transport specific miRNAs, proteins, and lncRNAs between cells, amplifying inflammatory and fibrotic signals and forming a vicious cycle. This mechanism is considered a significant factor in accelerating glomerulosclerosis and renal tubulointerstitial fibrosis (32, 33).

Under conditions of insulin resistance, exosomes originating from adipose tissue and other sources adversely affect podocytes and mesangial cells via specific miRNAs and mRNAs. Numerous studies have shown that circulating exosomes are linked to markers of renal injury associated with insulin resistance. For example, the level of WT1 mRNA in plasma extracellular vesicles positively correlates with the urinary albumin-to-creatinine ratio, whereas ACE mRNA shows a negative correlation. These altered expression profiles likely indicate the state of glomerular injury induced by insulin resistance (34). At the mesangial cell level, exosomes from glomerular mesangial cells exposed to high glucose levels are rich in miR-3147. This miRNA promotes mesangial cell proliferation and early apoptosis by targeting PRKAR2B, thereby accelerating the progression of DKD (20). These findings imply that exosomes from various sources collectively impact podocytes and mesangial cells in an insulin-resistant state, perpetuating a detrimental cycle.

Lipotoxicity intensifies renal tubulointerstitial fibrosis by modifying the lipid composition and miRNA profile of exosomes. Disorders in lipid metabolism significantly drive the progression of DKD. The lipid components within exosomes not only indicate lipid toxicity but also actively contribute to fibrosis. A study employing plasma extracellular vesicle (EV) metabolomics revealed notable alterations in metabolites such as sphingosine 1-phosphate, lysophosphatidylcholine (LPC(O-18:1/0:0)), and uracil in the plasma EVs of DKD patients. The combined detection of these lipid metabolites offers excellent predictive value for early DKD (35). Furthermore, miRNAs like miR-21 and miR-29, along with lncRNAs in urinary exosomes, play regulatory roles in renal fibrosis. Lipotoxicity may influence the expression of these factors by affecting the lipid membrane fluidity of exosomes and the loading of specific miRNAs, thereby promoting renal tubulointerstitial fibrosis (24).

Metabolic memory induces long-term epigenetic reprogramming via exosomes, perpetuating renal injury. This phenomenon, where persistent damage occurs despite later glycemic control, is due to epigenetic information carried by extracellular vesicles. Urinary exosomes provide a real-time reflection of pathological kidney changes. miRNAs, lncRNAs, and proteins within these exosomes not only contribute to inflammation and fibrosis but may also transmit the “memory” of hyperglycemia to downstream cells through epigenetic mechanisms such as DNA methylation and histone modification (24). Furthermore, transcriptome integration and Mendelian randomization analysis have identified two extracellular vesicle-related biomarkers, CMAS and RGS10, whose expression changes are linked to the pathogenesis of DKD and may be influenced by metabolic memory (36).

### 3.2 Role of circulatory exosomes in systemic damage in DKD

Exosomes in the circulatory system are essential carriers of intercellular communication and play a significant role in the pathogenesis and development of DKD. These exosomes can transport metabolic and inflammatory signals from other tissues to the kidneys, thereby initiating DKD and systemic vascular complications. Exosomes in the blood of diabetic patients may originate from activated immune cells, endothelial cells, or platelets, and exhibit significant alterations in their molecular cargo. For example, exosomes isolated from the serum of patients with diabetic retinopathy and nephropathy can induce dysfunction in human glomerular endothelial cells. This mechanism of action is associated with the upregulation of the coagulation factor fibrinogen α subunit and the downregulation of 1-methylhistidine (37). These findings indicate that circulatory exosomes may contribute to diabetic endothelial damage by affecting metabolic pathways. Changes in the miRNA profile in exosomes, which are stable carriers of noncoding RNA, are particularly noteworthy. It has been demonstrated that noncoding RNAs, including miRNAs, are key regulators of renal pathophysiology and that their dysregulation is a hallmark of kidney diseases such as DKD (38). In the context of DKD, specific miRNAs are transmitted intercellularly through exosomes, thereby regulating gene expression in target cells. For example, miR-483-5p in renal tubular epithelial cells can be sorted into exosomes via a mechanism mediated by heterogeneous nuclear ribonucleoprotein A1, after which it is secreted extracellularly and enters the urine. This process reduces the inhibitory effect of intracellular miR-483-5p on its target genes (such as MAPK1 and TIMP2), ultimately promoting extracellular matrix deposition and renal interstitial fibrosis (39). These findings indicate that miRNAs carried by circulating (or urinary) exosomes can serve as biomarkers, while the sorting and release processes themselves are also an active pathogenic mechanism. In addition, exosomes derived from kidney cells, such as glomerular mesangial cells, may also enter the circulation and exert systemic effects. In the DKD model, the expression of secreted frizzled- related protein 2 by glomerular mesangial cells increases, elevating SFRP2 protein levels in the blood, which in turn inhibits Wnt signaling. Inhibition of the Wnt pathway affects osteoblast differentiation and ultimately contributes to osteoporosis (40). This process suggests that the kidneys can release circulatory factors via vesicular carriers such as exosomes, thereby affecting bone health and forming the so-called “kidney–bone axis”. Collectively, these findings indicate that exosomes in circulation function as a complex platform for signal transmission. They not only reflect the state of systemic metabolic disorders in diabetes but also actively transmit signals related to metabolic memory, inflammation, and fibrosis remotely to the kidneys and other organs (41, 42). This process plays a central role in the mechanisms underlying systemic damage caused by DKD.

## 4 Therapeutic exosomes: a new strategy for intervention in diabetic kidney disease

### 4.1 Renal protective mechanism and preclinical efficacy evidence of therapeutic exosomes

Mesenchymal stem cell-derived exosomes (MSC-Exos) are emerging as a promising cell-free therapy for DKD (43). These nanovesicles are abundant in bioactive molecules, including growth factors, cytokines, and miRNAs, which are crucial for the paracrine functions of mesenchymal stem cells (44). Research indicates that MSC-Exos deliver significant anti-inflammatory, anti-apoptotic, and anti-fibrotic effects through specific miRNAs like miR-146a-5p, miR-424-5p, and miR-22-3p (45). For example, miR-146a-5p can reduce renal inflammation by modulating the TRAF6 and STAT1 signaling pathways and promoting macrophage polarization toward the anti-inflammatory M2 phenotype (45). miR-424-5p targets YES-associated protein1 (YAP1), preventing high glucose-induced apoptosis in renal tubular epithelial cells and epithelial-mesenchymal transition (EMT) (46). Additionally, miR-22-3p inhibits NLRP3 inflammasome activation, lowering inflammatory factors like IL-1β and IL-18, thereby protecting podocytes. Animal studies have confirmed its protective effects (47).

Animal studies have demonstrated the protective role of MSC-derived exosomes (MSC-Exos) in kidney health. In a rat model of streptozotocin- induced diabetic nephropathy, human umbilical cord MSC-derived exosomes (UC-MSC-exo) significantly reduce renal injury and inhibit both epithelial-to-mesenchymal transition (EMT) and renal fibrosis. This effect is linked to the suppression of the Hedgehog/SMO signaling pathway (48). Additionally, MSC-Exos mitigate apoptosis by inhibiting the p53 pathway through the delivery of microRNAs such as miR-125b-5p, and they decrease inflammatory responses by affecting the NOD2 signaling pathway (49). These diverse mechanisms collectively underpin the ability of MSC-Exos to improve proteinuria, renal function, and ameliorate histopathological damage in the DKD model (50).

A recent meta-analysis of 17 preclinical studies systematically assessed the efficacy of MSC-Exos. The findings revealed that MSC-Exos treatment significantly improved renal function parameters in animal models of diabetic nephropathy compared to the control group (51). Notably, the treatment effectively reduced the urine protein/creatinine ratio (ACR), with a standardized mean difference (SMD) of -2.00 (95% CI: -3.15 to -0.85), and significantly decreased serum creatinine (SMD = -1.45, 95% CI: -2.14 to -0.76) and blood urea nitrogen (SMD = -2.06, 95% CI: -3.01 to -1.11) (49). These results strongly support the efficacy of MSC-Exos in reducing proteinuria and enhancing renal function. The mechanism involves multiple effects, including anti-inflammation, anti-fibrosis, anti-apoptosis, and the induction of autophagy.

### 4.2 Application prospects of therapeutic exosomes

Natural exosomes face challenges, including inadequate targeting, low drug loading efficiency, and a brief *in vivo* half-life. Once administered intravenously, they are swiftly cleared by the mononuclear phagocytic system, particularly in the liver and spleen, which restricts their dosage and efficacy in reaching target tissues such as the kidneys (52). Consequently, enhancing exosome targeting and therapeutic effects through engineering modifications has become a significant research focus. Strategies involve pretreating parent cells, altering exosome membranes, or modifying their contents (52). For example, genetic engineering can express targeting peptides, such as RGD peptides, or antibodies on exosome surfaces, enabling precise targeting of specific cell types like podocytes in DKD (53). In various kidney disease models, modified exosomes have demonstrated significant therapeutic potential (54). In acute kidney injury models, they deliver the super inhibitor of NF-κB (srIκB), down- regulates the NF-κB signaling pathway to reduce inflammation and apoptosis, thereby enhancing renal function (55). In chronic kidney disease, they inhibit pro-fibrotic factors like TGF-β by delivering anti-fibrotic miRNAs, such as miR-29, which decreases extracellular matrix deposition (56). In diabetic nephropathy, miR-218-5p can be targeted and delivered to podocytes to activate mitochondrial autophagy, alleviating damage (54). Furthermore, siRNA can be delivered to silence RBM15, reducing fibrosis, inflammation, and senescence in glomerular mesangial cells (57).

**Figure 4:**
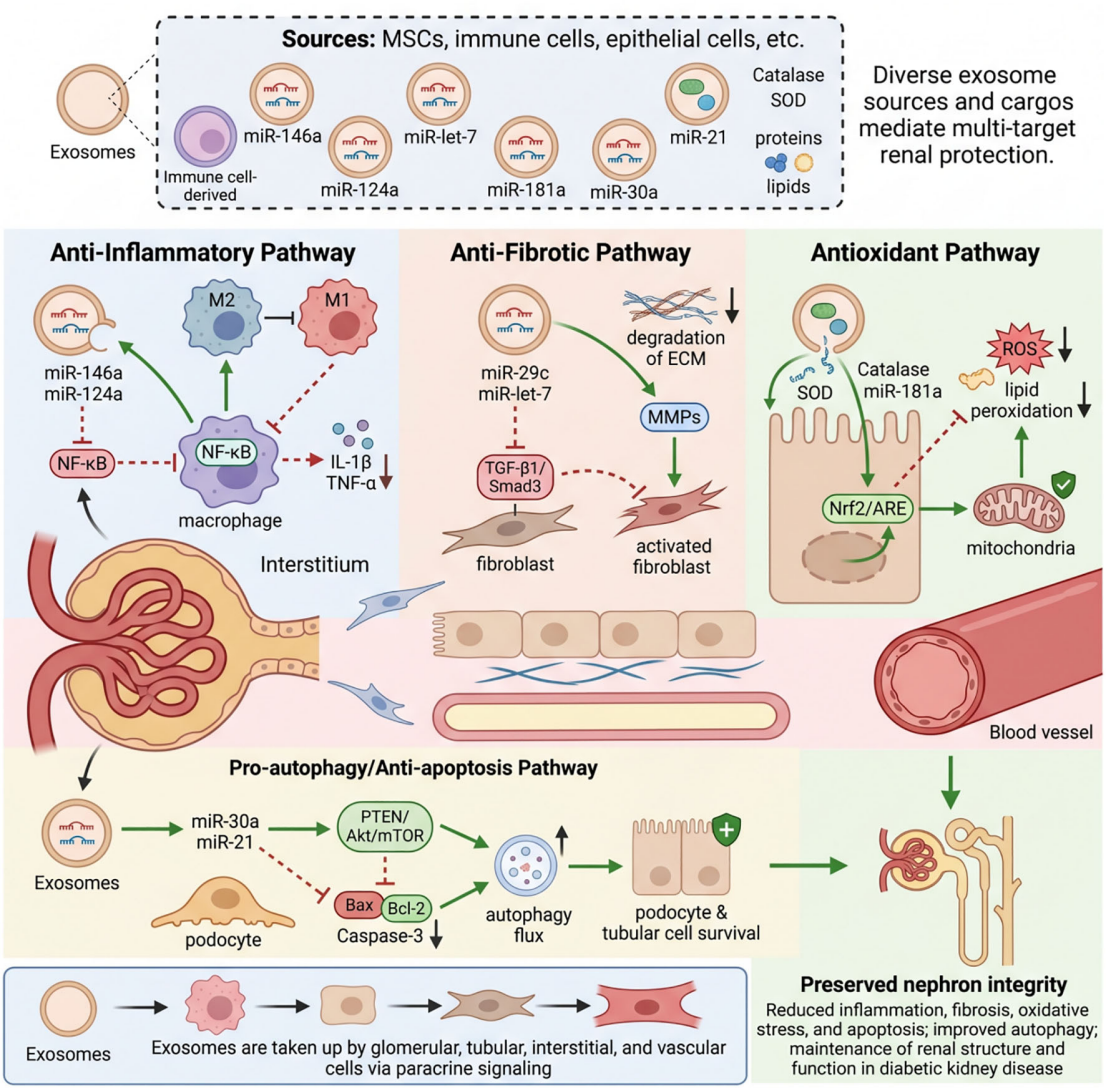
Therapeutic exosomes from different sources exert multi-target protective effects in the renal microenvironment of DKD, with major mechanisms encompassing anti-inflammatory effects, anti-fibrotic effects, alleviation of oxidative stress, and enhancement of autophagy and inhibition of apoptosis. Regarding anti-inflammatory mechanisms, molecules such as miR-146a and miR-124a carried by exosomes inhibit the NF-κB signaling pathway, reduce M1 macrophage polarization and the release of pro-inflammatory factors (IL-1β, TNF-α), and promote M2 polarization to alleviate renal interstitial inflammation. In anti-fibrosis, exosomes deliver miR-29c or miR-let-7 to target and inhibit the TGF-β1/Smad3 pathway, reduce fibroblast activation and collagen deposition, while upregulating matrix metalloproteinases to promote extracellular matrix degradation, delaying glomerulosclerosis and renal interstitial fibrosis. In alleviating oxidative stress, antioxidant enzymes (such as Catalase, SOD) or miR-181a contained in exosomes activate the Nrf2/ARE pathway, reducing levels of reactive oxygen species (ROS) and lipid peroxides, and protecting mitochondrial function. In improving autophagy and anti-apoptosis, exosomes regulate the PTEN/Akt/mTOR axis through miR-30a or miR-21 to restore impaired autophagic flux, while inhibiting Bax and Caspase-3 activity and upregulating Bcl-2, reducing apoptosis of podocytes and renal tubular epithelial cells, thereby maintaining nephron integrity. The schematic diagram illustrates that exosomes, secreted via paracrine signaling, are taken up by target cells in the glomerulus, tubulointerstitium, and vascular regions; dashed boxes indicate their diverse sources and key cargo molecules (miRNAs, proteins, lipids) acting as mediators of these protective effects.

The primary potential of modified exosomes is their capacity for precise modification to enable kidney-specific targeted delivery (**Figure 4**). Future developments are anticipated to include exosomes targeting podocytes, renal tubular epithelial cells, or diseased endothelial cells. This will allow the specific enrichment of therapeutic substances, such as anti-fibrotic miRNAs or anti- inflammatory cytokines, within the kidneys, thereby enabling precise regulation of local gene expression (58). Targeting core pathological processes like oxidative stress, inflammation, and fibrosis offers a novel strategy for advancing interventions in DKD (59). Additionally, modified exosomes have shown significant promise in promoting tissue regeneration and repair, as evidenced in osteochondral regeneration (60). This advancement offers hope for future therapies aimed at reversing nephron damage in DKD.

## 5 Meta-analysis evidence of the use of exosomes as biomarkers for DKD

### 5.1 The potential of urinary exosomal miRNA as biomarkers for DKD

In recent years, multiple studies have systematically evaluated the diagnostic value of urinary exosomal microRNA (miRNA) in distinguishing patients with DKD from those with diabetes alone or from healthy individuals. Urinary exosomal miRNAs originate from renal cells and are not only highly stable but also conveniently obtainable via noninvasive methods, making them a focus in early DKD diagnosis. RT-qPCR analysis revealed that the expression levels of miR-145-5p and miR-27a-3p in urinary exosomes from DKD patients were significantly higher than those in those from patients with diabetes alone and healthy controls (61). Another study revealed that urinary exosomal miR-615-3p was upregulated in DKD patients and that its levels were positively correlated with serum cystatin C, plasma TGF-β1, creatinine, urea nitrogen, and 24-hour urinary protein levels, whereas negatively correlated with estimated glomerular filtration rate (eGFR) and albumin levels, indicating a potential close association with renal inflammation and fibrosis processes (62). Furthermore, findings reveal that urinary exosomal miR-4534 is upregulated in DKD patients and is associated with microalbuminuria levels, potentially affecting the FoxO signaling pathway by targeting BNIP3, highlighting its potential that it may serve as a novel biomarker for DKD progression (63).

Summary analyses indicate that some urinary exosomal miRNAs have high diagnostic accuracy. For example, urinary exosomal miR-145-5p had an area under the receiver operating characteristic curve (AUC) of 0.88 for diagnosing DKD, indicating strong discriminatory ability (54). Another study confirmed that combining urinary exosomal miR-615-3p with the urinary albumin/creatinine ratio (ACR) yielded better diagnostic performance than using ACR alone, demonstrating its potential for noninvasive, early diagnosis of DKD (62). The stability of these miRNAs stems from their encapsulation within the exosomal lipid bilayer membrane, which protects them from ribonuclease degradation in urine, allowing them to remain intact in bodily fluids (64).

However, there are significant discrepancies among existing studies, posing challenges for clinical translation. The role of exosomes in DKD, their potential as biomarkers, and their therapeutic applications have garnered considerable attention. Some studies have specifically screened for stably expressed reference miRNAs in urinary exosomes to serve as internal reference genes for detecting biomarkers of kidney diseases (65). For example, one study aimed to establish a set of reliable reference genes for the molecular diagnosis of kidney diseases using urinary exosomes. Researchers analyzed 31 urine samples using microarray technology and identified 14 stably expressed miRNAs, 10 of which showed potential as reference genes-highlighting the necessity of establishing standardized internal references (65). Therefore, to translate these findings from the laboratory to the clinic, establishing standardized protocols for exosome isolation and miRNA detection is critical.

### 5.2 Integration of evidence on the association between blood exosome components and the progression of DKD

In recent years, an increasing number of studies have examined the association between exosomal components in plasma or serum and the stages of DKD or the rate of renal function decline, providing potential dynamic biomarkers for predicting disease progression. Circulating exosomes act as “messengers” between cells, and the substances they carry, such as proteins, miRNAs, and circular RNAs (circRNAs), can accurately reflect the pathophysiological state of their source cells and play crucial roles in DKD development (11). Previous studies have indicated that the expression level of miR-4449 in serum exosomes from patients with DKD is significantly elevated. These exosomes can exacerbate oxidative stress and pyroptosis through the miR-4449/HIC1 pathway, thereby aggravating kidney injury (66). Another study revealed that in exosomes released by mesangial cells treated with high glucose, the expression of the circular RNA circ_DLGAP4 is upregulated. This circRNA can act as a “sponge” to absorb miR-143 and promote mesangial cell proliferation and fibrosis by regulating the ERBB3/NF-κB/MMP-2 axis, thereby accelerating DKD progression (67).

Recent evidence suggests that the levels of specific molecules in circulating exosomes (such as certain miRNAs) are significantly correlated with renal function indicators and hold promise as dynamic markers for predicting disease progression. Data suggest that the expression level of miR-1207-5p in serum exosomes is reduced in patients with type 2 DKD, and this reduction is more pronounced in patients at higher risk of disease progression (68). Correlation analysis revealed that miR-1207-5p levels are negatively correlated with indicators of renal dysfunction (e.g., decreased eGFR and increased proteinuria), and multivariate logistic regression analysis indicated that it may be protective against DKD progression. ROC curve analysis further confirmed that exosomal miR-1207-5p can effectively discriminate among low-, high-, and very high-risk DKD patients (68). Additionally, mRNAs within extracellular vesicles in the blood have been explored as potential biomarkers. A cross-sectional study examined the expression of WT1 and ACE mRNA in blood extracellular vesicles from DKD patients, finding that WT1 mRNA was upregulated while ACE mRNA was downregulated. Both expression levels were significantly correlated with the urine albumin-to-creatinine ratio (ACR) and demonstrated high diagnostic accuracy for predicting overt DKD (AUCs of 0.83 and 0.75, respectively) (34).

The systematic integration of these findings indicates that changes in the circulating levels of both exosomal miRNAs and mRNAs are closely related to the severity and risk of DKD progression. These molecules play critical roles in core pathological processes of DKD, such as inflammation, oxidative stress, and fibrosis (69).

Exosomes derived from urine and blood, along with the miRNAs, mRNAs, and proteins they carry, demonstrate significant potential for the early detection, staging, progression monitoring, and treatment response evaluation in diabetic nephropathy (70) (**Table 1**). These findings offer valuable resources for developing new non-invasive or minimally invasive diagnostic tools. However, most current studies remain in the discovery and validation phases. Future research should prioritize large-scale, multicenter prospective cohorts to further validate the predictive value of these markers. Additionally, it is essential to explore their efficacy in conjunction with existing clinical indicators, such as eGFR and ACR, to achieve more precise risk stratification of diabetic nephropathy and to provide tailored management strategies for individual patients.

**Table 1:** Clinical significance of urinary and circulating exosomal components as candidate biomarkers for DKD.

Clinical function	Candidate biomarker (source)	Specific application evidence
Early diagnosis/detection	Urinary exosomal miR-320c, miR-6068	Significantly upregulated in microalbuminuric DN patients, correlated with albuminuria levels, serving as potential early predictive markers (70).
Early diagnosis/detection	Urinary exosomal miR-142-3p	Expression is elevated in DKD patients, shows good AUC in ROC analysis, and is positively correlated with UACR (71).
Early diagnosis/detection	Urinary exosomal proteins PAK6, EGFR	The combination showed an AUC of 0.912 for diagnosing DKD (0.897 in validation cohort), negatively correlated with eGFR and positively with Scr (72).
Early diagnosis/detection	Urinary exosomal protein regucalcin (SMP30)	Significantly downregulated in both DKD tissue and urinary exosomes, potentially reflecting early renal lesions (73).
Disease staging/severity	Urinary exosomal miR-188-5p, miR-150-3p, etc. (10 miRNAs)	Significantly upregulated (~13–15 fold) or downregulated (~7–9 fold) in biopsy-proven DKD patients with nephrotic-range proteinuria, associated with pathological severity (74).
Disease staging/severity	Serum exosomal miR-1246, let-7c-5p, etc. (8 miRNAs)	Specifically upregulated in DKD patients, and expression levels are significantly positively correlated with the degree of albuminuria (75).
Prognostic assessment	Urinary exosomal mRNA WT1	Can predict the rate of eGFR decline over subsequent years, reflecting podocyte injury, useful for risk stratification (76).
Prognostic assessment	Urinary exosomal protein Elf3	Detected only in urinary exosomes of DKD patients; its level closely correlates with the rate of eGFR decline (R²=0.726), indicating irreversible podocyte injury (77).
Progression monitoring	Urinary exosomal let-7c-5p	Negatively correlated with eGFR (r=-0.723), changes with DKD progression, with an AUC of 0.818 (78).
Progression monitoring	Urinary exosomal miR-92a-1-5p	Derived from proximal tubular epithelial cells, elevated in DKD, and can predict kidney injury and disease progression (30).
Diagnosis/Differentiation	Urinary exosomal proteins AMBP, MLL3, VDAC1	Differentially expressed in urinary exosomes from DKD patients validated by SRM, capable of distinguishing DKD from healthy controls (79).
Diagnosis/Differentiation	Urinary exosomal miR-29c-5p, miR-15b-5p	Downregulated in DKD, with AUCs of 0.774 and 0.818, respectively, for predicting DKD (80).

### 5.3 Clinical significance of exosome biomarkers in DKD

Urine and blood-derived exosomes are promising biomarkers for DKD, differing notably in stability, non-invasiveness, and disease specificity. Urine exosomes (uEVs) are particularly advantageous due to their high disease specificity. They primarily originate from kidney cells, such as renal tubular epithelial cells and podocytes, enabling them to more accurately reflect local kidney pathophysiological changes (69). For example, alterations in podocyte marker WT1 mRNA and renal tubular injury marker NGAL in urine exosomes are directly linked to glomeruli and renal tubule damage, offering a non-invasive detection advantage (81). However, urine exosome stability is susceptible to factors like urine pH, osmotic pressure, and sample processing. Standardizing separation and purification techniques, such as ultracentrifugation and dialysis, remains challenging, with different methods potentially affecting analysis outcomes (82). Conversely, plasma/serum exosomes offer greater sample stability and have more established pretreatment processes in clinical testing (83). These exosomes reflect vesicles released by cells throughout the body, including the kidneys, potentially providing broader disease state information. However, their disease specificity is lower because kidney signals may be diluted or obscured by vesicles from other organs (83).

The diagnostic performance of single markers versus multi-marker panels differs significantly in their application strategies. Single markers, like urine exosomal miR-192, are cost-effective and straightforward to detect, effectively identifying diabetic patients in the microalbuminuria stage (84). However, their sensitivity and specificity are often limited, failing to capture the complex, heterogeneous pathological processes of DKD (36). In contrast, multi-marker panels enhance diagnostic sensitivity and specificity by integrating data from various pathways. For example, a metabolomics study demonstrated that a combination of four metabolites-uracil, LPC(O-18:1/0:0), sphingosine1-phosphate, and 4-acetylamino- butyric acid-in plasma exosomes outperformed any single metabolite in predicting early DKD (35). Additionally, research combining transcriptome and Mendelian randomization analyses identified two exosome- related genes, CMAS and RGS10, as biomarkers, offering fresh perspectives on DKD management (36). Consequently, multi-marker panels more accurately identify DKD patients across different stages and pathological features (85).

Exosome-derived markers offer distinct advantages over traditional indicators like ACR and eGFR in early detection and dynamic monitoring of kidney conditions. In early detection, these markers can reveal molecular-level abnormalities in the kidneys sooner (**Table 2**). For example, α1-antitrypsin (α1-AT) levels in urine exosomes rise significantly before microalbuminuria appears in diabetic patients (86). Similarly, CCL21 mRNA levels in urine exosomes increase even when DKD patients have normal renal function (eGFR), effectively distinguishing early DKD cases (87). These insights provide an earlier opportunity for clinical intervention. In dynamic monitoring and understanding disease mechanisms, ACR and eGFR primarily indicate renal function outcomes. In contrast, exosomes carry active molecules like miRNA and mRNA, which are directly involved in disease pathogenesis. For instance, elevated levels of miR-21 and miR-29c in urine exosomes are closely linked to renal fibrosis (88). Tracking these markers not only monitors disease progression but also provides insight into active pathological mechanisms such as inflammation, fibrosis, and extracellular matrix deposition (83). Exosome markers are also valuable in identifying non-albuminuric DKD. Approximately 20-30% of DKD patients exhibit normal ACR despite decreased eGFR, limiting the diagnostic value of traditional indicators (89). Exosome markers, reflecting the renal cell state, are poised to become effective tools for identifying these patients, addressing the limitations of current diagnostic criteria (89).

**Table 2:** Comparison of extracellular vesicle biomarkers and conventional indicators in DKD.

Comparison dimension	Urinary extracellular vesicle (uEV) biomarkers	Plasma/serum extracellular vesicle biomarkers	Conventional indicators (ACR/eGFR)
Source & acquisition	Directly derived from renal cells (e.g., podocytes, tubular epithelial cells), enabling completely non-invasive sampling (69).	Derived from a mixture of cells throughout the body, requiring minimally invasive (blood draw) sampling (83).	Functional parameters calculated from urine or blood tests.
Stability	Can be influenced by urine physicochemical properties (pH, osmolality). Isolation and purification methods require standardization (82).	Relatively more stable, with pre-analytical processing being more established in clinical laboratories (83).	Highly stable, with well-standardized detection methods.
Disease specificity	High, as they directly mirror local pathological changes within the kidney (69).	Moderate to Low, as renal-derived signals may be diluted or interfered with by EVs from other organs (83).	High for renal function assessment, but cannot distinguish specific etiologies of kidney disease.
Detection time window	Very Early, with abnormalities potentially appearing before significant structural damage or functional decline (e.g., pre-microalbuminuria) (87).	Relatively Early, may precede some functional changes (35).	Late, typically manifesting after structural injury has occurred.
Mechanistic insight	Rich, as they carry functional molecules (nucleic acids, proteins) directly involved in disease pathogenesis (e.g., fibrosis, inflammation) (88).	Provides information on systemic pathophysiological states.	Lacking, as they reflect functional outcomes without indicating underlying molecular mechanisms.
Diagnostic performance (Sensitivity/Specificity)	Single markers (e.g., miR-192) show diagnostic value but may have limited sensitivity or specificity (83).	Single markers (e.g., AEBP1 mRNA) have shown diagnostic potential.	As individual indicators, ACR and eGFR have acceptable specificity but insufficient sensitivity for early-stage DKD, particularly in non-albuminuric phenotypes. They also lack granularity for risk stratification (89).
	Multi-marker panels (combining multiple miRNAs, mRNAs, or proteins) integrate information from different pathological pathways, demonstrating superior diagnostic sensitivity and specificity for accurately identifying patients at different disease stages (36).	Multi-marker panels (e.g., metabolite combinations) exhibit significantly better predictive power than individual markers, offering more robust diagnostic performance (35).	
Main advantages	Non-invasive, high renal specificity, suitable for early detection and mechanistic studies; multi-marker strategies can optimize diagnostic efficacy.	Sample stability, potential to reflect systemic-kidney crosstalk; multi-marker panels enhance diagnostic accuracy.	Routine clinical use, low cost, and clear interpretation of results.
Main limitations	Complex isolation/analysis techniques lacking standardization; susceptible to urine sample variables; diagnostic performance of single markers may be suboptimal.	Weaker kidney-specific signal; complex cellular origins.	Diagnosis is often delayed; inability to identify non-albuminuric DKD (80); insensitive to early pathological changes.

Although numerous studies have highlighted the diagnostic potential of exosome candidate markers in diabetic nephropathy, the existing evidence remains limited. Firstly, the sample sizes in these studies are typically small, which may result in insufficient statistical power and an overestimation of effect sizes. Secondly, there is considerable heterogeneity among the studies. The types of markers vary widely, including PAK6/EGFR protein, WT1, and miR-130a. Additionally, exosome separation methods differ, such as ultracentrifugation, PEG precipitation, and size exclusion chromatography. The research subjects also vary, covering patients with both type 1 and type 2 diabetes, and the stages of the disease are inconsistent. These discrepancies complicate the comparison and synthesis of results. Thirdly, there is a lack of external validation. Much of the evidence regarding stem cell exosomal miRNAs is derived from animal models rather than clinical settings. Furthermore, most markers have undergone validation only within single centers, lacking confirmation in independent, multi-center cohorts. This raises concerns about the generalizability of the findings. Furthermore, the issue of repeatability is significant. For example, although the WT1 protein has been independently reported by multiple research groups, its diagnostic threshold and detection standardization remain unestablished. Current research on exosome biomarkers is still in its early stages. These findings offer a promising direction for the early diagnosis of DKD. However, future research requires large-scale, multi-center, prospective cohort studies, and the adoption of a unified standard for exosome isolation and detection to confirm the reliability and practicality of clinical applications.

## 6 Safety assessment of exosome therapy

Existing research highlights extracellular vesicles (EVs), particularly exosomes, as promising therapeutic targets or vectors due to their pivotal role in DKD pathology (90). Therapeutic strategies focus on three main areas: inhibiting pathological EV generation, modifying their contents, and engineering them as therapeutic carriers. Each strategy’s clinical potential is closely tied to its specific safety considerations (91).The safety assessment of various intervention strategies focuses on distinct aspects. Firstly, inhibiting the production and release of pathological exosomes aims to block the transmission of injury signals. For instance, the exosome secretion inhibitor GW4869 can prevent the excretion of the intact membrane protein C-megalin in renal tubular cells, which is induced by albumin modified by advanced glycation end products (92). However, a primary safety concern associated with this strategy is that EV biogenesis is a ubiquitous physiological process. Extensive inhibition might disrupt normal intercellular communication and tissue homeostasis, necessitating careful evaluation of long-term systemic effects in complex *in vivo* models (92). Secondly, modifying exosome contents to regulate cellular functions focuses on precise intervention. Research indicates that knocking down pathogenic miR-221 in podocyte-derived EVs can reverse the dedifferentiation of renal tubular epithelial cells in diabetic mice (93). Similarly, reducing miR-21-5p in extracellular vesicles (sEVs) from renal tubular cells under high glucose conditions can counteract its promotion of epithelial-mesenchymal transition (91).

The safety of this strategy hinges on the specificity and precision of gene intervention. It is crucial to target only pathogenic molecules without disrupting other physiological RNA networks, which depends heavily on an efficient and specific delivery system. Lastly, modified exosomes as therapeutic carriers have a strong safety foundation. Preclinical studies show that camel milk-derived exosomes and human umbilical cord mesenchymal stem cell-derived exosomes (hucMSC-Exos) can enhance renal function and reduce oxidative stress and inflammation in DKD animal models, with no significant toxicity or adverse effects reported (94). For example, hucMSC-Exos promote macrophage polarization toward the M2 anti-inflammatory phenotype (94). These natural carriers offer good biocompatibility and low immunogenicity. However, the standardization of preparation processes, *in vivo* distribution and metabolism, and the long-term fate after engineering modifications (such as drug loading and targeted modification) still require systematic safety evaluation (95).

In conclusion, current exosome-based therapies for DKD show promise in preclinical studies, with safety assessments differing by approach. Inhibiting secretion necessitates evaluating overall impact, while content modification relies on precise targeting. Engineered natural vectors benefit from a stronger biological safety foundation (94). Future advancements in separation techniques, engineering strategies, and comprehensive safety evaluations, including long-term toxicology, are anticipated to enhance the safety and efficacy of exosome therapies for DKD.

## 7 Challenges and future directions: the path toward clinical translation

### 7.1 Establishment of technical standardization and regulatory frameworks

The translation of extracellular vesicles from basic research to clinical applications faces multiple challenges (**Table 3**). Two core difficulties are the lack of unified technical standards and the urgent need to establish regulatory frameworks. Regardless of whether biomarkers for disease diagnosis or therapeutic products are used, the standardization of methods for the isolation, purification, identification, and quantification of extracellular vesicles is a cornerstone for ensuring result reliability and product consistency (96). However, in reality, from plasma and serum to urine, and from ultracentrifugation to size-exclusion chromatography, isolation methods are diverse, and a universally recognized “gold standard” is still lacking (97). This absence of unified standards directly leads to significant variability in research data. For example, the reported concentration of extracellular vesicles in the plasma of healthy individuals can vary by orders of magnitude across studies, ranging from 4.50×10^8 to 6.70×10^11 particles per milliliter (98). Such large discrepancies not only make studies difficult to replicate but also severely hinder the development of diagnostic reagents and therapeutic products based on extracellular vesicles (96).

**Table 3:** Technical variability, standardization issues, and translational bottlenecks in exosome research for diabetic nephropathy.

Process step	Major sources of variability	Specific impact on biomarker research	Impediment to clinical translation	Mitigation strategies
Sample collection & pre-processing	Differences in collection tubes, anticoagulants, storage temperature, freeze-thaw cycles, and storage duration (104).	Affects EV yield, integrity, and cargo composition, causing baseline shifts in biomarker quantification and preventing direct comparison of results across studies or centers (104).	Hinders the establishment of a unified, robust clinical standard operating procedure (SOP), compromising the reproducibility of test results across different times and locations.	Develop and strictly adhere to unified sample collection guidelines (e.g., from ISEV) and meticulously document and report all pre-analytical steps (104).
EV isolation & purification	Lack of a “gold standard”; common methods (e.g., ultracentrifugation, size-exclusion chromatography, polymer-based precipitation) capture different EV subpopulations (exosomes, microvesicles) with varying purity (105).	EV preparations obtained by different methods are heterogeneous in concentration, size, and cargo (proteins, RNA) (106), leading to unstable biomarker discovery results and difficulty attributing signals to specific vesicle subtypes versus co-isolated contaminants (107).	The diagnostic performance of a candidate biomarker may vary dramatically across studies using different isolation methods, severely hindering its clinical validation and the development of standardized assay kits.	Employ complementary isolation methods (e.g., ultracentrifugation combined with size-exclusion chromatography) (106) and rigorously characterize and report the isolates according to MISEV guidelines (104).
EV characterization & quantification	Expression levels of commonly used markers (e.g., CD9, CD63, CD81) vary significantly between individuals and disease states (108). Instrument settings and analysis parameters differ for quantification methods like nanoparticle tracking analysis (NTA).	Over-reliance on a single tetraspanin marker may miss or misrepresent EVs from specific sources (108); particle count does not necessarily correlate with functional cargo (e.g., specific mRNA or protein) content.	Makes it difficult to establish a unified “dosing” standard (based on particle number, protein amount, or cargo molecule count), affecting the threshold setting for biomarkers and the dose standardization for therapeutic EVs (108).	Use multiparameter characterization (e.g., protein arrays) (108), report both qualitative (markers) and quantitative (e.g., total protein, specific RNA copy number) metrics, and report multiple quantification indices.
Cargo analysis (protein/mRNA/miRNA)	High-background proteins (e.g., in plasma) severely interfere with EV proteomic analysis. Technical variations in RT-qPCR, such as choice of reference genes and RNA extraction efficiency, are significant (34).	Low-abundance but disease-specific proteins or mRNAs (e.g., WT1, ACE, CDH2, MCP-1) may be masked or not stably detected (34). miRNA signatures identified in different studies can be conflicting or inconsistent.	The specificity and sensitivity of candidate biomarkers (e.g., urinary exosomal CCL21 mRNA (87)) are difficult to replicate in independent validation cohorts, preventing regulatory approval as a diagnostic tool.	Employ effective EV enrichment prior to mass spectrometry (109); standardize nucleic acid extraction and reference gene selection; conduct large-scale, multi-center prospective validation studies (34).

Faced with this dilemma, both academic and industrial communities are taking active measures. The International Society for Extracellular Vesicles (ISEV) has introduced the “Minimal Information for Studies of Extracellular Vesicles” (MISEV) guidelines and established data-sharing platforms such as EV-TRACK to promote standardized research practices and improve data reproducibility (96). In parallel, scientists are exploring the development of artificial exosomes or biomimetic particles as standard reference materials to provide standardized benchmarks for comparing and optimizing different methods (99). The regulatory landscape is also in an exploratory phase. Agencies such as the U.S. Food and Drug Administration (FDA) and the European Medicines Agency (EMA) are gradually developing evaluation frameworks for extracellular vesicle-based therapeutic products, including exosomes (100). A fundamental challenge lies in defining how these novel products should be classified, whether as biological products, advanced therapy medicinal products (ATMPs), or medical devices (101). For example, in Europe, exosome therapies are typically regulated as ATMPs (102). Regulatory authorities require manufacturers to provide comprehensive, standardized data covering the entire process, from cell source and culture conditions to exosome isolation, characterization, and even drug loading, to clearly define the product’s characteristics, quality, safety, and efficacy (101).Furthermore, existing tracking techniques (such as radioiodine labelling) may impair the functional integrity of exosomes (especially those derived from mesenchymal stem cells) (103). This reminds us that the development of nondestructive, highly sensitive novel labelling and *in vivo* tracking technologies has become an urgent priority. Only in this way can we accurately delineate the transport and metabolic patterns of exosomes *in vivo*.

Several other challenges affecting the translation of laboratory methods into real-world applications must also be acknowledged. For example, performance can vary between different production batches of commercial reagent kits, and the calibration status of automated instruments may introduce additional variability. Moreover, organizing large-scale prospective multicenter validation studies is both costly and difficult to coordinate, which often leads to a paucity of high-level clinical evidence.

In summary, for exosomes to truly benefit patients, they must overcome the “last mile” from the laboratory to the bedside. This requires establishing standardized operating procedures throughout the entire R&D process, defining a clear and feasible regulatory pathway, developing automated and integrated platforms for verification, and promoting multi-center collaborative research. This is the necessary path for turning dreams into reality.

### 7.2 Personalized medicine and combined therapy strategies

In the future, exosomes are anticipated to play a pivotal role in the management of DKD through the integration of personalized medicine and combination therapy. The essence of personalized diagnosis and treatment lies in precise diagnosis and risk stratification based on patient-specific exosome profiles. Research has identified distinct molecular “fingerprints” in the urinary exosomes of DKD patients. For example, DKD patients exhibit unique miRNA expression profiles, such as miR-320c and miR-30d-5p, which differ significantly from those of healthy individuals and patients with uncomplicated diabetes (70). Furthermore, a study involving biopsy-confirmed DKD cases identified specific miRNAs exhibiting differential regulation (upregulation or downregulation) in urinary exosomes (74). At the proteomic level, proteins like PAK6 and EGFR are found to be upregulated in the urinary exosomes of DKD patients, correlating with renal function indicators and suggesting their potential as diagnostic biomarkers (71). These discoveries provide a foundation for disease classification and diagnosis of DKD using exosome molecular profiles. However, there is a lack of prospective clinical evidence on applying these exosome characteristics to guide personalized treatment decisions, such as selecting targeted drugs or tailoring treatment plans for individual patients in DKD. This concept, inspired by liquid biopsy and precision medicine in oncology (91), remains largely speculative in the context of DKD.

In therapeutic contexts, combining exosome therapy with standard treatments offers promising synergy. Current standard treatments for diabetic kidney disease (DKD) include SGLT2 inhibitors, GLP-1 receptor agonists, and non-steroidal mineralocorticoid receptor antagonists. These drugs significantly interact with exosomes. Previous studies have shown that SGLT2 inhibitors delay DKD tubular lesions by inhibiting Hhip-positive extracellular vesicles (EVs) (110). The link between mineralocorticoid receptor antagonists and exosomes is based on the mechanism where MR activation causes abnormal EV release (111). Research on GLP-1 receptor agonists has not yet fully explored their direct regulation of the pro- inflammatory/anti-inflammatory factor balance in exosomes. However, the renal protective effects of GLP-1 receptor agonists may influence exosomes indirectly. Future studies should aim to elucidate the precise relationship between these standard therapies and the exosome regulatory network, potentially identifying new targets for the precise treatment of DKD.

In dermatology, exosomes are used for treatment, aesthetic applications, and regeneration, showing significant potential (112). However, direct evidence for their effectiveness in DKD primarily comes from preclinical studies. For example, research indicates that combining Balanites aegyptiacae plant extract with MSCs or their derived exosomes can synergistically reduce oxidative stress and inflammation in the DKD rat model, demonstrating superior efficacy compared to monotherapies (113).

Emerging research focuses on using exosome-delivered gene editing tools, like CRISPR-Cas9, to correct genetic defects associated with DKD. Modified exosomes are promising as safe and efficient carriers for the CRISPR-Cas9 ribonucleoprotein complex (114). Studies have shown success in delivering the CRISPR-Cas9 system to target cells via modified exosomes, enabling specific gene editing, such as the oncogene KRAS (115). While exosome-mediated CRISPR-Cas9 technology has shown revolutionary potential in cancer gene editing, particularly targeting oncogenes and tumor suppressor genes (116), its application in DKD remains theoretical. It could edit genes responsible for podocyte injury, renal tubular epithelial cell transdifferentiation, or abnormal fibrosis. However, direct research in DKD is lacking, with current inferences drawn from other diseases. To achieve clinical application, challenges like targeted delivery efficiency, off-target effects, and large-scale production must be addressed.

In conclusion, integrating exosome biomarker-based diagnosis, existing drug applications, and precise gene-editing interventions offers a promising future for DKD treatment. However, achieving this vision requires a comprehensive understanding of current evidence limitations and further research for confirmation.

## 8 Conclusion

**Figure 5:**
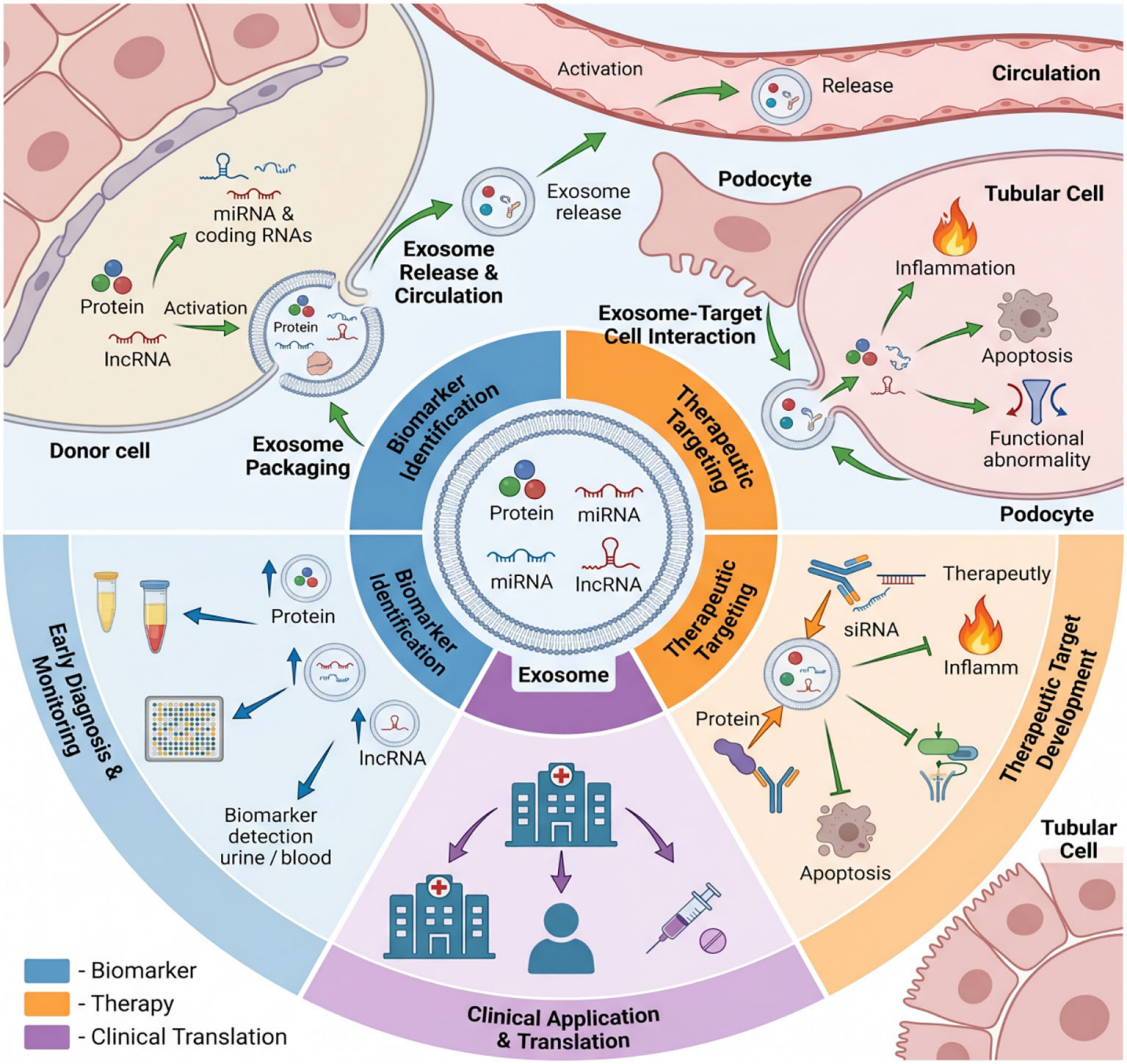
This figure illustrates the interrelationships among the pathogenic mechanisms, biomarkers, therapeutic applications, and clinical translation of exosomes in DKD. In the figure, podocytes and renal tubular epithelial cells serve as the core cellular sources, where donor cells package molecules such as miRNA, lncRNA, proteins, and siRNA to form exosomes. These exosomes are released into the circulation (bloodstream or urine), activating target cells (e.g., podocytes or renal tubular cells) through intercellular interactions, thereby inducing pathological processes including inflammation, apoptosis, and functional abnormalities. Concurrently, exosomes and their cargo—including miRNA, lncRNA, and proteins—detected in urine or blood can serve as biomarkers for early disease diagnosis. From a therapeutic perspective, exosomes function as delivery vehicles for therapeutic RNA or proteins, exerting anti-inflammatory and anti-apoptotic effects. Collectively, these mechanisms, biomarkers, and therapeutic strategies represent a translational pathway from fundamental research to clinical application. Distinct color blocks correspond to the four major themes: pathogenic mechanisms, biomarkers, therapeutic applications, and clinical translation.

Research on the role of exosomes in DKD has evolved from early descriptive observations to a core subject with profound pathological implications and broad translational prospects (**Figure 5**). From a professional perspective, their role has shifted from “pathogenic vectors” to “therapeutic hope,” which not only reflects a more in-depth understanding of the disease but also foreshadows profound changes in the diagnostic and therapeutic landscape. Existing studies confirm that, as key messengers in intercellular communication, exosomes undergo alterations in the composition and secretion patterns of their cargo (e.g., miRNAs and proteins) under high-glucose conditions, thereby transmitting proinflammatory and profibrotic signals that accelerate glomerulosclerosis and tubulointerstitial injury. Confirmation of this “pathogenic vector” identity provides a new theoretical entry point for intervening in disease progression.

In terms of diagnosis, meta-analyses targeting specific components (such as certain miRNA combinations and protein biomarkers) in urinary and bloodstream exosomes have contributed robust evidence to the field. These biomarkers demonstrate superior potential compared with traditional indicators for the early detection of DKD and for predicting disease progression. This represents the first step of exosome research, moving from mechanistic exploration to clinical practice-achieving noninvasive, dynamic clinical monitoring. However, experts also note that inconsistencies remain across different studies regarding biomarker selection, detection method standardization, and population differences. Therefore, future efforts still require large-scale, multicenter prospective studies to further validate their sensitivity and specificity and to establish unified operational guidelines and diagnostic thresholds.

In the therapeutic field, meta-analyses of preclinical studies have consistently demonstrated that mesenchymal stem cell-derived extracellular vesicles (MSC-EVs) significantly improve renal function, alleviate oxidative stress, and inhibit inflammation and fibrosis in animal models of DKD. This lays a solid scientific foundation for their potential as a new “cell-free therapy” strategy. It is widely recognized that the therapeutic value of MSC-EVs stems from their inherent dual characteristics: they can deliver various active substances to regulate target cell functions while possessing low immunogenicity and good biocompatibility. However, when various perspectives are synthesized, it is essential to fully recognize the differences between animal models and human disease, as well as the lack of unified standards in existing research regarding administration methods, dosing, and exosome preparation purity-all of which may affect the stability and comparability of therapeutic efficacy.

Transforming exosomes from “therapeutic hope” into mature clinical products in the future still comes with multiple challenges. Technically, it is necessary to establish a system for the large-scale production and quality control of exosomes with high purity and activity. From a scientific perspective, it is essential to elucidate their homing characteristics, metabolism, and long-term safety *in vivo*. At the regulatory level, developing evaluation pathways suitable for such novel biological therapeutic products is needed. Therefore, subsequent research should focus on several key directions. First, developing modified exosomes through surface modifications to achieve kidney targeting or loading of specific therapeutic molecules to enhance efficacy. Second, actively advancing rigorous early clinical trials to evaluate the safety and preliminary effects in patients with DKD at various stages. Third, exploring synergistic or combination strategies of exosome therapy with existing standard treatments (such as SGLT2 inhibitors and RAAS blockers) to maximize therapeutic outcomes.

In summary, the dual roles of exosomes in DKD have opened new avenues for understanding this disease and innovating its diagnosis and treatment. Although the translational path is challenging, sustained interdisciplinary collaboration and deep integration of basic research, technological development, and clinical validation may ultimately enable exosome-based approaches to deliver their promise of bringing new diagnostic and therapeutic options, that are more precise and effective, to patients with DKD.

## Glossary

DKD: Diabetic kidney disease
ESRD: End-Stage Renal Disease
RAAS: Renin–Angiotensin–Aldosterone System
ARB: Angiotensin II Receptor Blocker
SGLT2: Sodium–Glucose Cotransporter 2
LAMP2A: Lysosomal-Associated Membrane Protein 2A
TGF-β: Transforming Growth Factor Beta
MAPK: Mitogen-Activated Protein Kinase
TIMP2: Tissue Inhibitor of Metalloproteinases 2
NLRP3: NOD-, LRR- and pyrin domain-containing protein 3
EMT: Epithelial–Mesenchymal Transition
DLGAP4: Discs Large Homolog-Associated Protein 4
ERBB3: Erb-B2 Receptor Tyrosine Kinase 3
NF-κB: Nuclear Factor Kappa B
MMP-2: Matrix Metalloproteinase-2
WT1: Wilms Tumor 1
BNIP3: BCL2/adenovirus E1B 19 kDa Protein-Interacting Protein 3
MSCs: Mesenchymal Stem/Stromal Cells
MSC-EVs/MSC-Exos: Mesenchymal Stem Cell–Extracellular Vesicles/Exosomes
eGFR: estimated Glomerular Filtration Rate
ACR: Albumin-to-Creatinine Ratio
ATMPs: Advanced Therapy Medicinal Products
PAK6: p21-Activated Kinase 6
EGFR: Epidermal Growth Factor Receptor
CRISPR-Cas9: Clustered Regularly Interspaced Short Palindromic Repeats–CRISPR Associated Protein 9

## References

[1] Cai R Li C Zhao Y Yuan H Zhang X Liang A . Traditional chinese medicine in diabetic kidney disease: Multifaceted therapeutic mechanisms and research progress. Chin Med. (2025) 20:95. doi: 10.1186/s13020-025-01150-w 40598250 PMC12211247

[2] Ma L Liu D Yu Y Li Z Wang Q . Immune-mediated renal injury in diabetic kidney disease: From mechanisms to therapy. Front Immunol. (2025) 16:1587806. doi: 10.3389/fimmu.2025.1587806 40534883 PMC12173918

[3] Wang Y Jin M Cheng CK Li Q . Tubular injury in diabetic kidney disease: Molecular mechanisms and potential therapeutic perspectives. Front Endocrinol. (2023) 14:1238927. doi: 10.3389/fendo.2023.1238927 37600689 PMC10433744

[4] Santos GL Santos CFD Rocha GR Calmon MS Lemos FF Silva LG . Beyond glycemic control: Roles for sodium-glucose cotransporter 2 inhibitors and glucagon-like peptide-1 receptor agonists in diabetic kidney disease. World J Diabetes. (2025) 16:104706. doi: 10.4239/wjd.v16.i6.104706 40548289 PMC12179897

[5] Tartau CG Boboc IKS Mititelu-Tartau L Bogdan M Buca BR Pavel LL . Exploring the protective effects of traditional antidiabetic medications and novel antihyperglycemic agents in diabetic rodent models. Pharm (Basel Switzerland). (2025) 18(5):670. doi: 10.3390/ph18050670 40430489 PMC12114790

[6] Baker LW Ovincy C Souvalian L Hickson LJ Chebib FT . Contemporary management of advanced chronic kidney disease: An evidence-based review. Eur J Internal Med. (2026) 143:106557. doi: 10.1016/j.ejim.2025.106557 41130867 PMC12560998

[7] Basuli D Kavcar A Roy S . From bytes to nephrons: AI’s journey in diabetic kidney disease. J Nephrol. (2025) 38:25–35. doi: 10.1007/s40620-024-02050-2 39133462 PMC11903625

[8] Fyfe J Casari I Manfredi M Falasca M . Role of lipid signalling in extracellular vesicles-mediated cell-to-cell communication. Cytokine Growth Factor Rev. (2023) 73:20–6. doi: 10.1016/j.cytogfr.2023.08.006 37648617

[9] Tang S Yu S Cheng J Zhang Y Huang X . The versatile roles and clinical implications of exosomal mRNAs and microRNAs in cancer. Int J Biol Markers. (2020) 35(2):3–19. doi: 10.1177/1724600820920293 32389046

[10] Nail HM Chiu C-C Leung C-H Ahmed MMM Wang H-M . Exosomal miRNA-mediated intercellular communications and immunomodulatory effects in tumor microenvironments. J BioMed Sci. (2023) 30:69. doi: 10.1186/s12929-023-00964-w 37605155 PMC10440907

[11] Han L Cai X Zhou H . Exosomal microRNAs: Potential nanotherapeutic targets for diabetic kidney disease. Nanomedicine (London England). (2023) 18:1669–80. doi: 10.2217/nnm-2023-0023 37909293

[12] Mani S Gurusamy N Ulaganathan T Paluck AJ Ramalingam S Rajasingh J . Therapeutic potentials of stem cell-derived exosomes in cardiovascular diseases. Exp Biol Med (Maywood NJ). (2023) 248:434–44. doi: 10.1177/15353702231151960 36740769 PMC10281619

[13] Aloi N Drago G Ruggieri S Cibella F Colombo P Longo V . Extracellular vesicles and immunity: At the crossroads of cell communication. Int J Mol Sci. (2024) 25:1205. doi: 10.3390/ijms25021205 38256278 PMC10816988

[14] Yıldızhan Y Bourke AM Jackson HK Manna PT Palmulli R Edgar JR . Exosome tethering requires tetherin homodimerisation. Biol Cell. (2025) 117:e70046. doi: 10.1111/boc.70046 41410165 PMC12712889

[15] Li M Li S Du C Zhang Y Li Y Chu L . Exosomes from different cells: Characteristics, modifications, and therapeutic applications. Eur J Med Chem. (2020) 207:112784. doi: 10.1016/j.ejmech.2020.112784 33007722

[16] Ferreira JV Ferraz LC Soares AR Ejtehadifar M Carvalho AS Hall MJ . LAMP2A regulates endosomal protein composition and membrane identity in exosome biogenesis. iScience. (2026) 29:114305. doi: 10.1016/j.isci.2025.114305 41503212 PMC12768870

[17] Adem B Bastos N Ruivo CF Sousa-Alves S Dias C Vieira PF . Exosomes define a local and systemic communication network in healthy pancreas and pancreatic ductal adenocarcinoma. Nat Commun. (2024) 15:1496. doi: 10.1038/s41467-024-45753-7 38383468 PMC10881969

[18] Novaes AS Felizardo RJF Camara NOS Agrawal S Boim MA . Extracellular vesicles facilitate the crosstalk between high glucose-stimulated mesangial cells and healthy podocytes to mediate injury responses. Int J Mol Sci. (2026) 27:1927. doi: 10.3390/ijms27041927 41752063 PMC12940433

[19] Han Q Zou Y Yang Q Ran E Li Z Liu F . CD4+ T-cell-derived small extracellular vesicles induce the apoptosis of renal tubular epithelial cells in diabetic nephropathy by regulating mitochondrial dynamics. Biochim Biophys Acta Mol Basis Dis. (2026) 1872:168131. doi: 10.1016/j.bbadis.2025.168131 41401562

[20] Zhang J Zhao F Tao Y Hu M Bai Y Li J . Glomerular mesangial derived extracellular vesicles deteriorate diabetic kidney disease via miR-3147/PRKAR2B axis. Ren Fail. (2025) 47:2519817. doi: 10.1080/0886022x.2025.2519817 40571682 PMC12203694

[21] Perico L Remuzzi G Benigni A . Mitochondria to the rescue: Organelle trafficking in renal health and disease. Nephron. (2026) 150:228–37. doi: 10.1159/000550092 41417683

[22] Li L Zheng Z Lan W Tang N Zhang D Ling J . Role of exosomes in cardiovascular disease: A key regulator of intercellular communication in cardiomyocytes. ACS Omega. (2025) 10:18145–69. doi: 10.1021/acsomega.4c11423 40385188 PMC12079207

[23] Perez-Hernandez J Riffo-Campos AL Ortega A . Urinary and plasma-derived exosomes reveal a distinct microRNA signature associated with albuminuria in hypertension. Hypertension. (2021) 77:960–71. doi: 10.1161/hypertensionaha.120.16598 33486986

[24] Haripriya V Anandh U Dinda AK Kalita B . Urinary exosomes in diabetic kidney disease: Pathophysiological insights and diagnostic potential. Life Sci. (2026) 399:124477. doi: 10.1016/j.lfs.2026.124477 42176882

[25] Zhou X Guo Z Zhang R Yan R . Proteome profiling reveals inflammation and fibrosis biomarkers in urinary migrasomes of patients with diabetic kidney disease. J Proteome Res. (2026) 25:2344–58. doi: 10.1021/acs.jproteome.5c01073 41906798

[26] Ji C Li B Zhang J Shi L Zhang L Shi H . Dual-targeted engineered mesenchymal stem cell-derived extracellular vesicles delivering Nedd4 attenuate renal fibrosis in diabetic kidney disease. Mater Today Bio. (2026) 38:103097. doi: 10.1016/j.mtbio.2026.103097 42006708 PMC13091060

[27] Navez M Detry O Jouret F . Mesenchymal/stromal cell therapy in kidney diseases: An update. Curr Opin Nephrol Hypertens. (2026) 35:465–72. doi: 10.1097/mnh.0000000000001184 41925211

[28] Wen J Ma Z Livingston MJ Zhang W Yuan Y Guo C . Decreased secretion and profibrotic activity of tubular exosomes in diabetic kidney disease. Am J Physiol Renal Physiol. (2020) 319:F664–73. doi: 10.1152/ajprenal.00292.2020 32715764 PMC7642884

[29] Zhu M Sun X Qi X Xia L Wu Y . Exosomes from high glucose-treated macrophages activate macrophages andinduce inflammatory responses via NF-κB signaling pathway *in vitro* and *in vivo*. Int Immunopharmacol. (2020) 84:106551. doi: 10.1016/j.intimp.2020.106551 32388490

[30] Tsai Y-C Kuo M-C Hung W-W Wu P-H Chang W-A Wu L-Y . Proximal tubule- derived exosomes contribute to mesangial cell injury in diabetic nephropathy via miR-92a-1-5p transfer. Cell Communication Signaling: CCS. (2023) 21:10. doi: 10.1186/s12964-022-00997-y 36639674 PMC9838003

[31] Yang H Bai Y Fu C Liu W Diao Z . Exosomes from high glucose-treated macrophages promote epithelial-mesenchymal transition of renal tubular epithelial cells via long non-coding RNAs. BMC Nephrol. (2023) 24:24. doi: 10.1186/s12882-023-03065-w 36717805 PMC9887774

[32] Thongboonkerd V . Roles for exosome in various kidney diseases and disorders. Front Pharmacol. (2019) 10:1655. doi: 10.3389/fphar.2019.01655 32082158 PMC7005210

[33] Thongboonkerd V Kanlaya R . The divergent roles of exosomes in kidney diseases: Pathogenesis, diagnostics, prognostics and therapeutics. Int J Biochem Cell Biol. (2022) 149:106262. doi: 10.1016/j.biocel.2022.106262 35787447

[34] Hashemi E Dehghanbanadaki H Baharanchi AA Forouzanfar K Kakaei A Mohammadi SM . WT1 and ACE mRNAs of blood extracellular vesicle as biomarkers of diabetic nephropathy. J Transl Med. (2021) 19:299. doi: 10.1186/s12967-021-02964-6 34246281 PMC8272332

[35] Pan Y Yang H Chen T Jin J Ruan L Hu L . Extracellular vesicles metabolic changes reveals plasma signature in stage-dependent diabetic kidney disease. Ren Fail. (2022) 44:1840–9. doi: 10.1080/0886022x.2022.2118067 36368309 PMC9662026

[36] Yang X Yue R Zhao L Wang Q . Integration of transcriptome and Mendelian randomization analyses in exploring the extracellular vesicle-related biomarkers of diabetic kidney disease. Ren Fail. (2025) 47:2458767. doi: 10.1080/0886022x.2025.2458767 39957315 PMC11834810

[37] Yang J Liu D Liu Z . Integration of metabolomics and proteomics in exploring the endothelial dysfunction mechanism induced by serum exosomes from diabetic retinopathy and diabetic nephropathy patients. Front Endocrinol. (2022) 13:830466. doi: 10.3389/fendo.2022.830466 35399949 PMC8991685

[38] Todorović P Pavlović N Maglica M Bajt P Kelam N Raguž F . Non-coding RNAs as emerging regulators in kidney pathophysiology: From molecular mechanisms to therapeutic potential. Genes. (2025) 16(11):1328. doi: 10.3390/genes16111328 PMC12652717 41300780

[39] Liu D Liu F Li Z Pan S Xie J Zhao Z . HNRNPA1-mediated exosomal sorting of miR-483-5p out of renal tubular epithelial cells promotes the progression of diabetic nephropathy-induced renal interstitial fibrosis. Cell Death Dis. (2021) 12:255. doi: 10.1038/s41419-021-03460-x 33692334 PMC7946926

[40] Xing H Liu Y Qu M Zhang Z Zeng Y Li P . Exosome-based Sfrp2 inhibition in mesangial cells alleviates osteoporosis and promotes osteointegration in diabetic kidney disease. Regener Biomater. (2025) 12:rbaf093. doi: 10.1093/rb/rbaf093 41030909 PMC12478701

[41] Zhu Y Liu C Hallajzadeh J . Understanding the roles of non-coding RNAs and exosomal non-coding RNAs in diabetic nephropathy. Curr Mol Med. (2025) 25:537–55. doi: 10.2174/0115665240287631240321072504 38591211

[42] Xu Y-X Pu S-D Li X Yu Z-W Zhang Y-T Tong X-W . Exosomal ncRNAs: Novel therapeutic target and biomarker for diabetic complications. Pharmacol Res. (2022) 178:106135. doi: 10.1016/j.phrs.2022.106135 35192956

[43] Liu L Chen Y Li X Wang J Yang L . Therapeutic potential: The role of mesenchymal stem cells from diverse sources and their derived exosomes in diabetic nephropathy. Biomedicine Pharmacotherapy = Biomedecine Pharmacotherapie. (2024) 175:116672. doi: 10.1016/j.biopha.2024.116672 38677249

[44] Barakat N Ali M Nassr A Zahran F . The potential role of exosome-derived mesenchymal stem cells and balanites aEgyptiaca in diabetic nephropathy amelioration in rats. Cell Mol Biol (Noisy-Le-Grand France). (2023) 69:37–44. doi: 10.14715/cmb/2023.69.2.7 37224048

[45] Zhang Y Le X Zheng S Zhang K He J Liu M . MicroRNA-146a-5p-modified human umbilical cord mesenchymal stem cells enhance protection against diabetic nephropathy in rats through facilitating M2 macrophage polarization. Stem Cell Res Ther. (2022) 13:171. doi: 10.1186/s13287-022-02855-7 35477552 PMC9044847

[46] Cui C Zang N Song J Guo X He Q Hu H . Exosomes derived from mesenchymal stem cells attenuate diabetic kidney disease by inhibiting cell apoptosis and epithelial-to- mesenchymal transition via miR-424-5p. FASEB Journal: Off Publ Fed Am Societies For Exp Biol. (2022) 36:e22517. doi: 10.1096/fj.202200488r 36036527

[47] Wang Y Liu J Wang H Lv S Liu Q Li S . Mesenchymal stem cell-derived exosomes ameliorate diabetic kidney disease through the NLRP3 signaling pathway. Stem Cells (Dayton Ohio). (2023) 41:368–83. doi: 10.1093/stmcls/sxad010 36682034

[48] Zhang K Zheng S Wu J He J Ouyang Y Ao C . Human umbilical cord mesenchymal stem cell-derived exosomes ameliorate renal fibrosis in diabetic nephropathy by targeting hedgehog/SMO signaling. FASEB Journal: Off Publ Fed Am Societies For Exp Biol. (2024) 38:e23599. doi: 10.1096/fj.202302324r 38572590

[49] Wang Y Lu D Lv S Liu X Liu G . Mesenchymal stem cell-derived exosomes ameliorate diabetic kidney disease through NOD2 signaling pathway. Renal Failure. (2024) 46:2381597. doi: 10.1080/0886022x.2024.2381597 39039856 PMC11268218

[50] Wang Y Shan S-K Guo B Li F Zheng M-H Lei L-M . The multi-therapeutic role of MSCs in diabetic nephropathy. Front Endocrinol. (2021) 12:671566. doi: 10.3389/fendo.2021.671566 34163437 PMC8216044

[51] Man X Lin T Xie Z Jin J He Q . Beneficial effects of cell-derived exosomes on diabetic nephropathy: A systematic review and meta-analysis of preclinical evidence. Acta Diabetologica. (2025) 62:607–20. doi: 10.1007/s00592-025-02473-8 39998649

[52] Choi H Choi Y Yim HY Mirzaaghasi A Yoo JK Choi C . Biodistribution of exosomes and engineering strategies for targeted delivery of therapeutic exosomes. Tissue Eng Regener Med. (2021) 18:499–511. doi: 10.1007/s13770-021-00361-0 34260047 PMC8325750

[53] Abbasi R Alamdari-Mahd G Maleki-Kakelar H Momen-Mesgin R Ahmadi M Sharafkhani M . Recent advances in the application of engineered exosomes from mesenchymal stem cells for regenerative medicine. Eur J Pharmacol. (2025) 989:177236. doi: 10.1016/j.ejphar.2024.177236 39753159

[54] Guo Z Gao S Wang Z Chen Z Chen J Duan A . Engineered RGD-Treg-Exos targeted delivery of miR-218-5p to activate mitophagy and attenuate podocyte injury in diabetic kidney disease. Adv Sci (Weinh). (2025) 12:e12034. doi: 10.1002/advs.202412034 40827657 PMC12499504

[55] Kim S Lee SA Yoon H Kim MY Yoo JK Ahn SH . Exosome-based delivery of super-repressor IκBα ameliorates kidney ischemia-reperfusion injury. Kidney Int. (2021) 100:570–84. doi: 10.1007/978-3-032-05988-8_268 34051264

[56] Li Y Waheed YA Sun D . Exosomes and renal fibrosis: Diagnostic value, therapeutic potential and challenges. Int J Nanomedicine. (2025) 20:11267–94. doi: 10.2147/ijn.s529311 40969664 PMC12442909

[57] Dong Q Dong L Zhu Y Wang X Yan X . Epigenetic silencing of MSTN via m6A modification underlies the renoprotective effects of engineered MSC exosomes with RBM15 depletion in diabetic nephropathy. Funct Integr Genomics. (2025) 25:244. doi: 10.1007/s10142-025-01746-3 41247540

[58] Wang L Wang D Ye Z Xu J . Engineering extracellular vesicles as delivery systems in therapeutic applications. Adv Sci (Weinh). (2023) 10:e2300552. doi: 10.1002/advs.202300552 37080941 PMC10265081

[59] Lu Y Wang S Yu B Li X . Engineered extracellular vesicles for treatment of inflammatory diseases. Mol Biotechnol. (2025). doi: 10.1007/s12033-025-01528-z 41252109

[60] Samadi P Sheykhhasan M Omer I Ullah A Zarea A Toomajian V . Regeneration of cartilage defects using engineered extracellular vesicles. Biofabrication. (2025). doi: 10.1088/1758-5090/addc41 40403758

[61] Han L-L Wang S-H Yao M-Y Zhou H . Urinary exosomal microRNA-145-5p and microRNA-27a-3p act as noninvasive diagnostic biomarkers for diabetic kidney disease. World J Diabetes. (2024) 15:92–104. doi: 10.4239/wjd.v15.i1.92 38313849 PMC10835498

[62] Wang J Tao Y Zhao F Liu T Shen X Zhou L . Expression of urinary exosomal miRNA-615-3p and miRNA-3147 in diabetic kidney disease and their association with inflammation and fibrosis. Renal Failure. (2023) 45:2121929. doi: 10.1080/0886022x.2022.2121929 36695327 PMC9879181

[63] Zhao Y Shen A Guo F Song Y Jing N Ding X . Urinary exosomal MiRNA-4534 as a novel diagnostic biomarker for diabetic kidney disease. Front Endocrinol. (2020) 11:590. doi: 10.3389/fendo.2020.00590 32982978 PMC7484971

[64] Zhang X Zhang K Fan Q Sang J Kan C Pan R . The role and therapeutic potential of exosome-mediated microRNAs regulatory networks in diabetic kidney disease. Mol Biol Rep. (2025) 52:902. doi: 10.1007/s11033-025-11015-y 40944788

[65] Mishra DD Maurya PK Tiwari S . Reference gene panel for urinary exosome-based molecular diagnostics in patients with kidney disease. World J Nephrol. (2024) 13:99–105. doi: 10.5527/wjn.v13.i3.99105 39351186 PMC11439094

[66] Gao C Wang B Chen Q Wang M Fei X Zhao N . Serum exosomes from diabetic kidney disease patients promote pyroptosis and oxidative stress through the miR-4449/HIC1 pathway. Nutr Diabetes. (2021) 11:33. doi: 10.1038/s41387-021-00175-y 34732690 PMC8566490

[67] Bai S Xiong X Tang B Ji T Li X Qu X . Exosomal circ_DLGAP4 promotes diabetic kidney disease progression by sponging miR-143 and targeting ERBB3/NF-κB/MMP-2 axis. Cell Death Dis. (2020) 11:1008. doi: 10.1038/s41419-020-03169-3 33230102 PMC7683700

[68] Zhou X Zhao J Wang J He K Du H You Q . Alterations in serum exosomal miR-1207-5p levels reflect severity and progression risk in type 2 diabetic kidney disease. BMC Nephrol. (2025) 26:440. doi: 10.1186/s12882-025-04360-4 40770290 PMC12329926

[69] Alli AA . Extracellular vesicles: Investigating the pathophysiology of diabetes-associated hypertension and diabetic nephropathy. Biol (Basel). (2023) 12:1138. doi: 10.3390/biology12081138 37627022 PMC10452642

[70] Delić D Eisele C Schmid R Baum P Wiech F Gerl M . Urinary exosomal miRNA signature in type II diabetic nephropathy patients. PloS One. (2016) 11:e0150154. doi: 10.1371/journal.pone.0150154 26930277 PMC4773074

[71] Li S Sun C Zheng S Lin S Li H Wu J . Expression and diagnostic value evaluation of urinary exosomal miR-142-3p in diabetic nephropathy. Sci Rep. (2025) 15:23991. doi: 10.1038/s41598-025-06002-z 40615506 PMC12227739

[72] Li T Liu TC Liu N Li MJ Zhang M . Urinary exosome proteins PAK6 and EGFR as noninvasive diagnostic biomarkers of diabetic nephropathy. BMC Nephrol. (2023) 24:291. doi: 10.1186/s12882-023-03343-7 37789280 PMC10548700

[73] Zubiri I Posada-Ayala M Benito-Martin A Maroto AS Martin-Lorenzo M Cannata-Ortiz P . Kidney tissue proteomics reveals regucalcin downregulation in response to diabetic nephropathy with reflection in urinary exosomes. Transl Res. (2015) 166:474–84.e4. doi: 10.1016/j.trsl.2015.05.007 26072307

[74] Lee WC Li LC Ng HY Lin PT Chiou TT Kuo WH . Urinary exosomal microRNA signatures in nephrotic, biopsy-proven diabetic nephropathy. J Clin Med. (2020) 9:1220. doi: 10.3390/jcm9041220 32340338 PMC7231152

[75] Kim H Bae YU Jeon JS Noh H Park HK Byun DW . The circulating exosomal microRNAs related to albuminuria in patients with diabetic nephropathy. J Transl Med. (2019) 17:236. doi: 10.1186/s12967-019-1983-3 31331349 PMC6647278

[76] Abe H Sakurai A Ono H Hayashi S Yoshimoto S Ochi A . Urinary exosomal mRNA of WT1 as diagnostic and prognostic biomarker for diabetic nephropathy. J Med Invest. (2018) 65:208–15. doi: 10.2152/jmi.65.208 30282862

[77] Sakurai A Ono H Ochi A Matsuura M Yoshimoto S Kishi S . Involvement of Elf3 on Smad3 activation-dependent injuries in podocytes and excretion of urinary exosome in diabetic nephropathy. PloS One. (2019) 14:e0216788. doi: 10.1371/journal.pone.0216788 31150422 PMC6544199

[78] Li W Yang S Qiao R Zhang J . Potential value of urinary exosome-derived let-7c-5p in the diagnosis and progression of type II diabetic nephropathy. Clin Lab. (2018) 64:709–18. doi: 10.7754/clin.lab.2018.171031 29739042

[79] Zubiri I Posada-Ayala M Sanz-Maroto A Calvo E Martin-Lorenzo M Gonzalez-Calero L . Diabetic nephropathy induces changes in the proteome of human urinary exosomes as revealed by label-free comparative analysis. J Proteomics. (2014) 96:92–102. doi: 10.1016/j.jprot.2013.10.037 24211404

[80] Tan J Chen Y Chuan F Gao Y Wu M Hu J . Integrative multi-omics suggests core gene dysregulation of histidine metabolism in diabetic kidney disease. Diabetes Med. (2026) 43:e70238. doi: 10.1111/dme.70238 41947261

[81] Ugarte F Santapau D Gallardo V Garfias C Yizmeyián A Villanueva S . Urinary extracellular vesicles as a source of NGAL for diabetic kidney disease evaluation in children and adolescents with type 1 diabetes mellitus. Front Endocrinol (Lausanne). (2022) 12:654269. doi: 10.3389/fendo.2021.654269 35046888 PMC8762324

[82] Barreiro K Dwivedi OP Leparc G Rolser M Delic D Forsblom C . Comparison of urinary extracellular vesicle isolation methods for transcriptomic biomarker research in diabetic kidney disease. J Extracell Vesicles. (2020) 10:e12038. doi: 10.1002/jev2.12038 33437407 PMC7789228

[83] Yu Y Shan Y Ding A Qian H . Extracellular vesicles in diabetic kidney disease: Emerging mechanisms, therapeutic implications, and biomarker prospects. Biochem Pharmacol. (2026) 244:117605. doi: 10.1016/j.bcp.2025.117605 41352566

[84] Jia Y Guan M Zheng Z Zhang Q Tang C Xu W . miRNAs in urine extracellular vesicles as predictors of early-stage diabetic nephropathy. J Diabetes Res. (2016) 2016:7932765. doi: 10.1155/2016/7932765 26942205 PMC4749815

[85] Ding L Li Z Xia Y Liu S Zhang M Liu L . Exosomes in diabetic kidney disease: Pathogenesis, biomarker discovery, and emerging therapeutics-a comprehensive systematic review. Ren Fail. (2026) 48:2640720. doi: 10.1080/0886022x.2026.2640720 41845912 PMC13003845

[86] Ning J Xiang Z Xiong C Zhou Q Wang X Zou H . Alpha1-antitrypsin in urinary extracellular vesicles: A potential biomarker of diabetic kidney disease prior to microalbuminuria. Diabetes Metab Syndr Obes. (2020) 13:2037–48. doi: 10.2147/dmso.s250347 32606862 PMC7306457

[87] Feng Y Zhong X Ni HF Wang C Tang TT Wang LT . Urinary small extracellular vesicles derived CCL21 mRNA as biomarker linked with pathogenesis for diabetic nephropathy. J Transl Med. (2021) 19:355. doi: 10.1186/s12967-021-03030-x 34404433 PMC8371892

[88] Hu M Shen X Zhou L . Role of extracellular vesicle-derived noncoding RNAs in diabetic kidney disease. Kidney Dis (Basel). (2024) 10:303–12. doi: 10.1159/000539024 39131883 PMC11309761

[89] Saenz-Pipaon G Echeverria S Orbe J Roncal C . Urinary extracellular vesicles for diabetic kidney disease diagnosis. J Clin Med. (2021) 10:2046. doi: 10.3390/jcm10102046 34064661 PMC8151759

[90] Chen J Zhang Q Liu D Liu Z . Exosomes: Advances, development and potential therapeutic strategies in diabetic nephropathy. Metabolism. (2021) 122:154834. doi: 10.1016/j.metabol.2021.154834 34217734

[91] Zhang M Lu Y Wang L Mao Y Hu X Chen Z . Autocrine small extracellular vesicles induce tubular phenotypic transformation in diabetic nephropathy via miR-21-5p. Gene. (2025) 938:149156. doi: 10.1016/j.gene.2024.149156 39653091

[92] De S Kuwahara S Hosojima M Ishikawa T Kaseda R Sarkar P . Exocytosis-mediated urinary full-length megalin excretion is linked with the pathogenesis of diabetic nephropathy. Diabetes. (2017) 66:1391–404. doi: 10.2337/db16-1031 28289043

[93] Su H Qiao J Hu J Li Y Lin J Yu Q . Podocyte-derived extracellular vesicles mediate renal proximal tubule cells dedifferentiation via microRNA-221 in diabetic nephropathy. Mol Cell Endocrinol. (2020) 518:111034. doi: 10.1016/j.mce.2020.111034 32926967

[94] Florijn BW Duijs JMGJ Levels JH Dallinga-Thie GM Wang Y Boing AN . Diabetic nephropathy alters the distribution of circulating angiogenic microRNAs among extracellular vesicles, HDL, and Ago-2. Diabetes. (2019) 68:2287–300. doi: 10.2337/db18-1360 31506344

[95] Zhao Z Yan Q Zhou S Liu F Liu Y Ren J . MYO1C is a urinary extracellular vesicle biomarker and mediator of podocyte injury in diabetic nephropathy. JCI Insight. (2026) 11:e194604. doi: 10.1172/jci.insight.194604 41569692 PMC13041675

[96] Yadav A Xuan Y Sen CK Ghatak S . Standardized reporting of research on exosomes to ensure rigor and reproducibility. Adv Wound Care. (2024) 13:584–99. doi: 10.1089/wound.2024.0093 38888007 PMC12344122

[97] Martins TS Vaz M Henriques AG . A review on comparative studies addressing exosome isolation methods from body fluids. Anal Bioanal Chem. (2023) 415:1239–63. doi: 10.1007/s00216-022-04174-5 35838769

[98] Mengual E-V Cordero L Pinto H . Human blood exosomes: Isolation and characterization methods, variability, and the need for standardized protocols-a review. Biomedicines. (2025) 13(12):2970. doi: 10.3390/biomedicines13122970 41462985 PMC12731227

[99] García-Manrique P Serrano-Pertierra E Lozano-Andrés E López-Martín S Matos M Gutiérrez G . Selected tetraspanins functionalized niosomes as potential standards for exosome immunoassays. Nanomaterials (Basel Switzerland). (2020) 10(5):971. doi: 10.3390/nano10050971 32443605 PMC7712311

[100] Li Q Li Y Shao J Sun J Hu L Yun X . Exploring regulatory frameworks for exosome therapy: Insights and perspectives. Health Care Sci. (2025) 4:299–309. doi: 10.1002/hcs2.70028 40861511 PMC12371722

[101] Wang C-K Tsai T-H Lee C-H . Regulation of exosomes as biologic medicines: Regulatory challenges faced in exosome development and manufacturing processes. Clin Transl Sci. (2024) 17:e13904. doi: 10.1111/cts.13904 39115257 PMC11307316

[102] Ansarian MA Fatahichegeni M Wang Y Ren J Zhang T Wang X . Exosomal biomarkers in leukemia: Translational potential and regulatory challenges for precision medicine applications. Front Immunol. (2025) 16:1677088. doi: 10.3389/fimmu.2025.1677088 41103412 PMC12521259

[103] Yang C-T Lai RC Phua VJX Aw SE Zhang B Sim WK . Standard radio-iodine labeling protocols impaired the functional integrity of mesenchymal stem/stromal cell exosomes. Int J Mol Sci. (2024) 25(7):3742. doi: 10.3390/ijms25073742 38612553 PMC11011818

[104] Ding X Wang X Du J Han Q Zhang D Zhu H . A systematic review and meta-analysis of urinary extracellular vesicles proteome in diabetic nephropathy. Front Endocrinol (Lausanne). (2022) 13:866252. doi: 10.3389/fendo.2022.866252 36034457 PMC9405893

[105] Gonçalves MO Di Iorio JF Marin GV Meneghetti P Negreiros NGS Torrecilhas AC . Extracellular vesicles. Curr Top Membr. (2024) 94:1–31. doi: 10.1111/resp.13756 39370203

[106] Shaabani N Meira SR Marcet-Palacios M Kulka M . Multiparametric biosensors for characterizing extracellular vesicle subpopulations. ACS Pharmacol Transl Sci. (2023) 6:387–98. doi: 10.1021/acsptsci.2c00207 36926451 PMC10012251

[107] Stahl PD Raposo G . Exosomes and extracellular vesicles: the path forward. Essays Biochem. (2018) 62:119–24. doi: 10.1042/ebc20170088 29765006

[108] Jørgensen M Bæk R Pedersen S Søndergaard EK Kristensen SR Varming K . Extracellular vesicle (EV) array: microarray capturing of exosomes and other extracellular vesicles for multiplexed phenotyping. J Extracell Vesicles. (2013) 2. doi: 10.3402/jev.v2i0.20920 PMC3760630 24009888

[109] Choi DS Kim DK Kim YK Gho YS . Proteomics of extracellular vesicles: exosomes and ectosomes. Mass Spectrom Rev. (2015) 34:474–90. doi: 10.1002/mas.21420 24421117

[110] Chang SY Liao MC Miyata KN Pang Y Zhao XP Peng J . Canagliflozin inhibits hedgehog interacting protein (Hhip) induction of tubulopathy in diabetic Akita mice. Transl Res. (2025) 277:13–26. doi: 10.1016/j.trsl.2024.12.005 39756674

[111] rOchiai-Homma F Kuribayashi-Okuma E Tsurutani Y Ishizawa K Fujii W Odajima K . Characterization of pendrin in urinary extracellular vesicles in a rat model of aldosterone excess and in human primary aldosteronism. Hypertens Res. (2021) 44:1557–67. doi: 10.1038/s41440-021-00710-5 34326480 PMC8645477

[112] Nahm WJ Nikas C Goldust M Horneck N Cervantes JA Burshtein J . Exosomes in dermatology: a comprehensive review of current applications, clinical evidence, and future directions. Int J Dermatol. (2025) 64:1995–2010. doi: 10.1111/ijd.17903 40533901

[113] Zahran F Nabil A Nassr A Barakat N . Amelioration of exosome and mesenchymal stem cells in rats infected with diabetic nephropathy by attenuating early markers and aquaporin-1 expression. Braz J Biol. (2023) 83:e271731. doi: 10.1590/1519-6984.271731 37466513

[114] Duan L Ouyang K Wang J Xu L Xu X Wen C . Exosomes as targeted delivery platform of CRISPR/Cas9 for therapeutic genome editing. ChemBioChem. (2021) 22:3360–8. doi: 10.1002/cbic.202100359 34418266

[115] McAndrews KM Xiao F Chronopoulos A LeBleu VS Kugeratski FG Kalluri R . Exosome-mediated delivery of CRISPR/Cas9 for targeting of oncogenic kras G12D in pancreatic cancer. Life Sci Alliance. (2021) 4(9e202000875.):. doi: 10.26508/lsa.202000875 34282051 PMC8321670

[116] Balaraman AK Babu MA Moglad E Mandaliya V Rekha MM Gupta S . Exosome-mediated delivery of CRISPR-Cas9: a revolutionary approach to cancer gene editing. Pathol Res Pract. (2025) 266:155785. doi: 10.1016/j.prp.2024.155785 39708520

