# Supplementary figures and images for: From pathogenic carriers to therapeutic hope: the dual role and translational prospects of exosomes in diabetic kidney disease
Source: Front Endocrinol (Lausanne). 2026 Jul 2;17:1831272. doi: 10.3389/fendo.2026.1831272 (PMC13372626; doi:10.3389/fendo.2026.1831272)

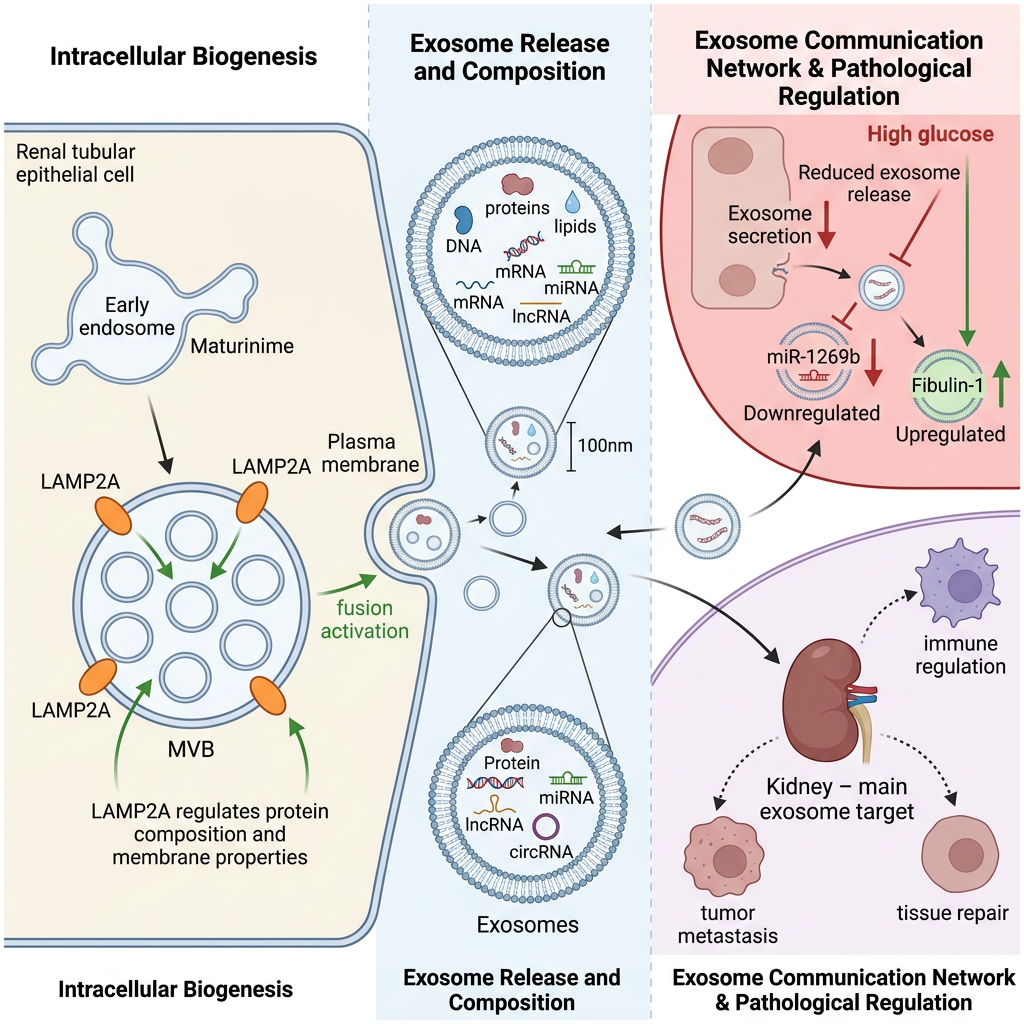

Supplement: Supplementary file 1 [file Image1.png]

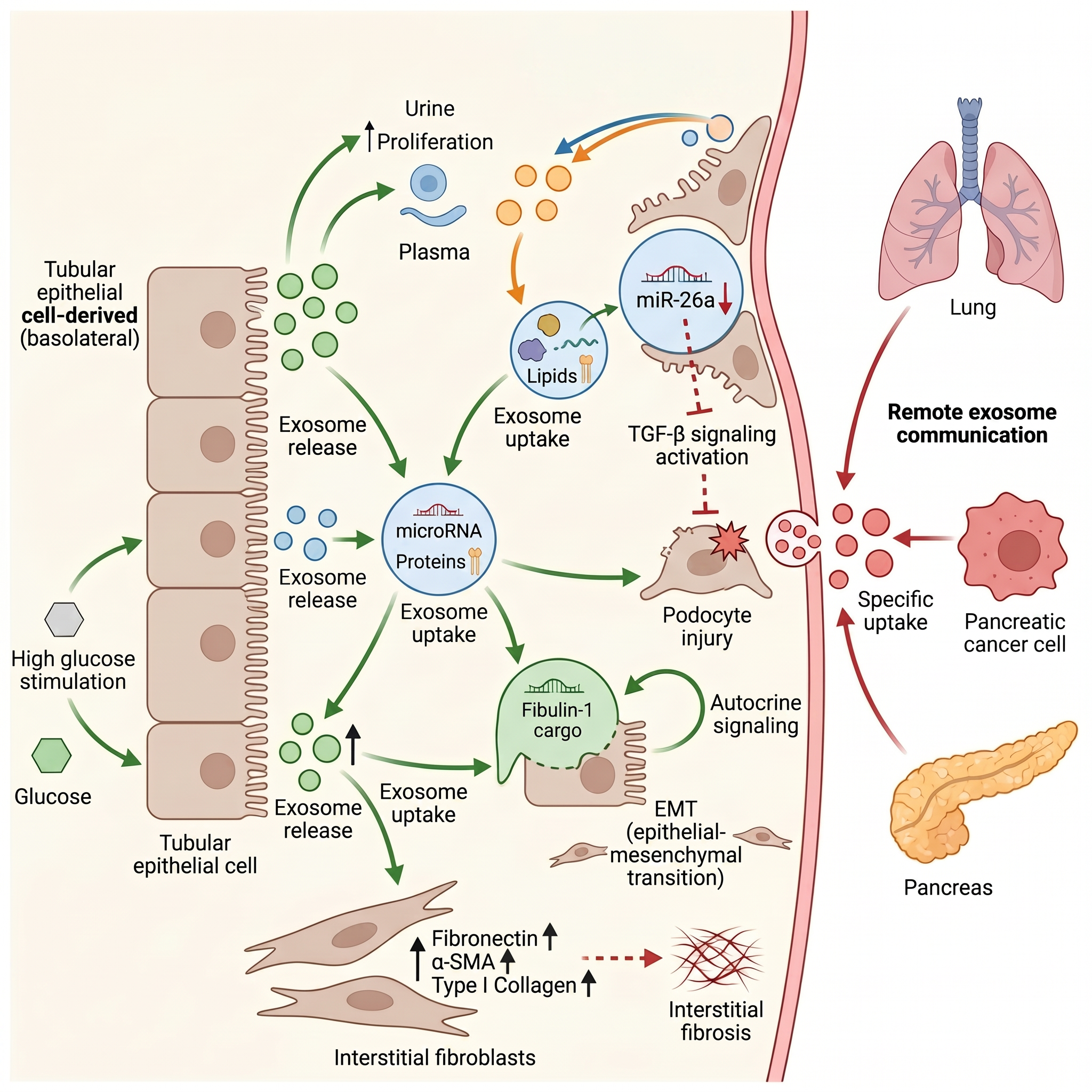

Supplement: Supplementary file 2 [file Image2.png]
